# Invasion Potential of *Calotropis procera* (Aiton) W.T. Aiton and *Xanthium strumarium* L. in the Anthropocene of Ethiopia: Implications for Management

**DOI:** 10.1002/ece3.72577

**Published:** 2025-12-09

**Authors:** Daniel Melese, Mulatu Ayenew Aligaz, Ahmed Seid Ahmed

**Affiliations:** ^1^ Department of Plant Biology and Biodiversity Management Addis Ababa University Addis Ababa Ethiopia; ^2^ Department of Biology Mizan‐Tepi University Tepi Ethiopia; ^3^ Department of Zoological Sciences Addis Ababa University Addis Ababa Ethiopia; ^4^ Department of Biology Debre Markos University Debre Markos Ethiopia; ^5^ Department of Biology Hawassa University Hawassa Ethiopia

**Keywords:** anthropocene, climate change, ensemble model, invasive species, management

## Abstract

The spread of invasive species presents a significant global challenge that threatens natural habitats, agricultural productivity, and biodiversity. In Ethiopia, Calotropis procera and Xanthium strumarium are aggressively invasive noxious weeds whose expansion is intensified by human activities and climate change. This study aimed to model the current and future distribution and habitat suitability of these species across Ethiopia to support effective management strategies. An ensemble modelling approach was employed, combining seven algorithms that include both machine learning and regression‐based methods, under various settings with ten replications. Predictions were made for current environmental conditions as well as future climate scenarios projected for the 2050s and 2070s. The results showed that human footprint and bioclimatic variables were the most influential predictors of habitat suitability for both species. Under current conditions, approximately 47,652 and 45,901 km^2^ were found suitable for Calotropis procera and Xanthium strumarium, respectively, showing a 40% overlap between their ranges. Under future climate projections, suitable habitats are expected to increase significantly, reaching up to 96,629 km^2^ by the 2070s for Calotropis procera and 57,121 km^2^ by the 2050s for Xanthium strumarium. These suitable areas are primarily located in the central, northern, southern, and eastern lowlands of Ethiopia and substantially overlap with biodiversity‐rich zones and major agricultural regions. The findings highlight that the expansion of both species is mainly driven by human influence, affecting large portions of Ethiopia's lowlands, midlands, and grasslands. To reduce further spread, it is important to implement targeted control efforts along roadsides, highways, and riverbanks, together with community‐based weeding campaigns that address various land use systems.

## Graphical abstract

The spread of invasive species poses significant challenge in Ethiopia aggravated by climate change and human activities.

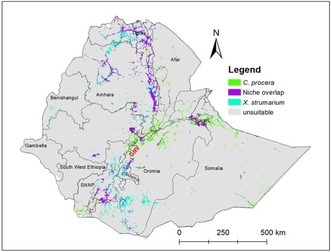

## 1 Introduction

The Anthropocene is a proposed geological epoch defined by the extensive and lasting influence of human activities on the Earth's natural systems. The mid‐20th century was marked by intensified industrialization, global trade, urban expansion, and land‐use change, which contributed to widespread environmental transformation (Head et al. 2022). These human‐driven forces have had a profound impact on biodiversity, leading to habitat degradation, species extinctions, and large‐scale disruptions to ecosystems and the climate (Head et al. 2022). One notable consequence of these changes is the accelerated spread of invasive plant species, which have taken advantage of the disturbed and interconnected landscapes characteristic of this human‐dominated era (Ricciardi et al. 2022). Additionally, because of anthropogenic pressures and climate change, many plant species are now migrating to higher mountains (Jump et al. 2012; Melese, Lemessa, et al. 2025), with animals being pushed to higher altitudes and potentially qualifying as climate refugees in Ethiopia (Chala et al. 2016; Ahmed, Bekele, et al. 2023; Kufa et al. 2025).

Invasive plant species pose significant threats to biodiversity by disrupting ecological processes, degrading ecosystem services, and affecting socioeconomic systems (Didham et al. 2005; McGeoch et al. 2010). In recent decades, their spread has greatly impacted local biodiversity, environmental quality, and ecosystem functioning (Rai and Singh 2020), often altering key ecological processes (Raizada et al. 2008). These species can reduce native species richness and abundance through mechanisms such as competition, predation, hybridization, and various indirect effects (Gaertner et al. 2009). They also change community composition (Hejda et al. 2009) and may influence the genetic diversity of native species (Schierenbeck and Ellstrand 2009).

Although biological invasions are known to interfere with ecosystem functioning and services, their effects on native species through trophic interactions and taxonomic disruptions remain underexplored on a global level (Mollot et al. 2017). They become a major driver of both ecological damage and economic losses across the world. In the United States, for instance, invasive plants rank as the second most significant threat to biodiversity after habitat destruction, contributing to annual losses of about $34 billion (Randall 1996; Pimentel et al. 2005). Similarly, in China, invasive alien species are responsible for yearly losses estimated at $14.45 billion, with more than 80% attributed to indirect impacts (Xu et al. 2006). In the European Union, a list of invasive alien species likely to threaten biodiversity and ecosystems has been developed, highlighting their potential negative impacts and identifying the most vulnerable biogeographic regions (Roy et al. 2019).

In Ethiopia, invasive species are considered the third leading cause of threats to biodiversity, economic losses, and public health problems (Shiferaw et al. 2018), following habitat degradation and the unsustainable exploitation of biological resources (Gebretsadik 2016). Currently, around 35 alien invasive species have been identified in the country (Shiferaw et al. 2018), and with the growing impacts of climate change, the risks associated with these species, including *Parthenium hysterophorus* L.and *Salvia tiliifolia* Vahl, are expected to intensify (Melese, Woldu, et al. 2025). In addition to their economic burden, invasive species also threaten native flora and fauna. For example, the rapid expansion of *Eichhornia crassipes* (Mart.) Solms (water hyacinth) in Lake Tana is an emerging concern in Ethiopia, where its impact is currently under investigation (Asmare et al. 2020).

**FIGURE 1:**
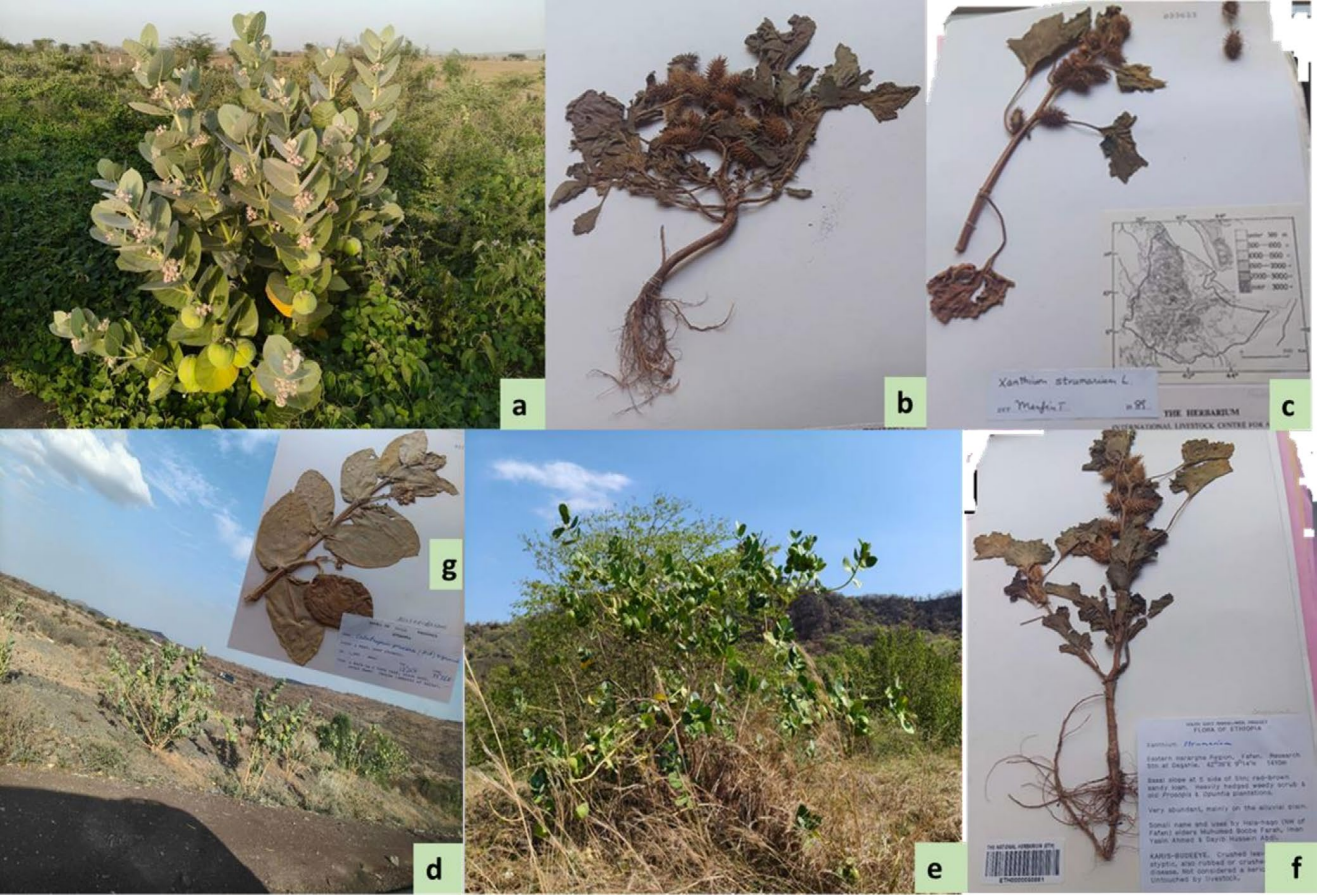
Invasive species *Calotropis procera* and *Xanthium strumarium* . (a, d) *Calotropis procera* invading along roadsides; (e) at the forest edge; (b) prepared sample of *Xanthium strumarium* ; (c, f) herbarium specimens of *Xanthium strumarium* ; (g) herbarium specimens of *Calotropis procera* used in the study (Photo Credit: ASA and DM).

*Calotropis procera* (Aiton) W.T Aiton and *Xanthium strumarium* L. are among the prominent invasive species in Ethiopia (Shiferaw et al. 2019). *C. procera* is a stout, succulent shrub, typically ranging from 2 to 7 m in height. It exhibits weak to strong branching, with young parts covered in a short tomentose layer that often gives a powdery (farinose) appearance, gradually becoming smooth as they mature. This species thrives in hot, arid environments, commonly occurring in dry riverbeds and other disturbed areas, from sea level up to 2250 m (Hedberg et al. 1996). Meanwhile, *X. strumarium* is an erect, robust annual or short‐lived perennial herb, generally 30–75 cm tall but capable of reaching up to 3 m along riverbanks. It has unarmed stems and typically invades farmlands, roadside ditches, and stream or riverbanks, occurring between 900 and 2000 m above sea level. It is cosmopolitan in distribution and well‐adapted to a wide range of disturbed habitats (Tadesse 2004). Like *C. procera* , it also exhibits allelopathic properties that suppress the growth of crops, native plants, and grasses (Gulzar and Siddiqui 2017; Seifu et al. 2024) (Figure 1).

Species distribution models (SDMs) have become essential tools for assessing how climate change influences the geographic spread and potential expansion of invasive plant species (Menge et al. 2016; Waheed et al. 2024). These models offer critical insights for the early detection of invasive weeds (Srivastava et al. 2019), support the development of rapid response mechanisms, and inform the design of effective control and eradication strategies under changing climate conditions scenarios (Pyke et al. 2008; Briscoe Runquist et al. 2019; Beaury et al. 2020; Melese, Woldu, et al. 2025). The advancement of SDMs in recent years has enabled researchers to predict the potential distribution patterns of invasive species more accurately (Mainali et al. 2015). In this study, we applied ensemble modeling approaches to predict the habitat suitability of two invasive species, *C. procera* and *X. strumarium* . Ensemble models, which combine multiple algorithms, are recognized for offering higher accuracy and robustness than single‐method models (Araújo and New 2007; Thuiller et al. 2016), and have been widely used to forecast both current and future spread of invasive plants (Stohlgren et al. 2010; Figuerola‐Ferrando et al. 2024).

To date, few studies have examined the invasion risk of *C. procera* and *X. strumarium* in different parts of Ethiopia (Erenso 2024; Amare et al. 2025). However, none have employed species distribution modeling (SDM) to predict their potential distribution. Consequently, this study provides an SDM‐based assessment of these invasive species in Ethiopia, addressing a key gap in understanding their ecological impacts and guiding management strategies. The objectives of this study were to (1) identify the most influential environmental variables determining the distribution of *C. procera* and *X. strumarium* , (2) map their current and projected suitable habitats for the years 2050 and 2070, and (3) assess the spatial and temporal shifts in their potential ranges to inform future weed management efforts. We hypothesize that (H1) climate change will increase the invasion potential of *C. procera* and *X. strumarium* by expanding the availability of suitable habitats, and (H2) human footprint variables will contribute more strongly to the modeling of these invasive species. The results of this study highlight areas in Ethiopia that are highly vulnerable to invasion, offering a scientific foundation for developing targeted management and mitigation strategies.

## 2 Methods

### 2.1 Species Occurrence Data

**FIGURE 2:**
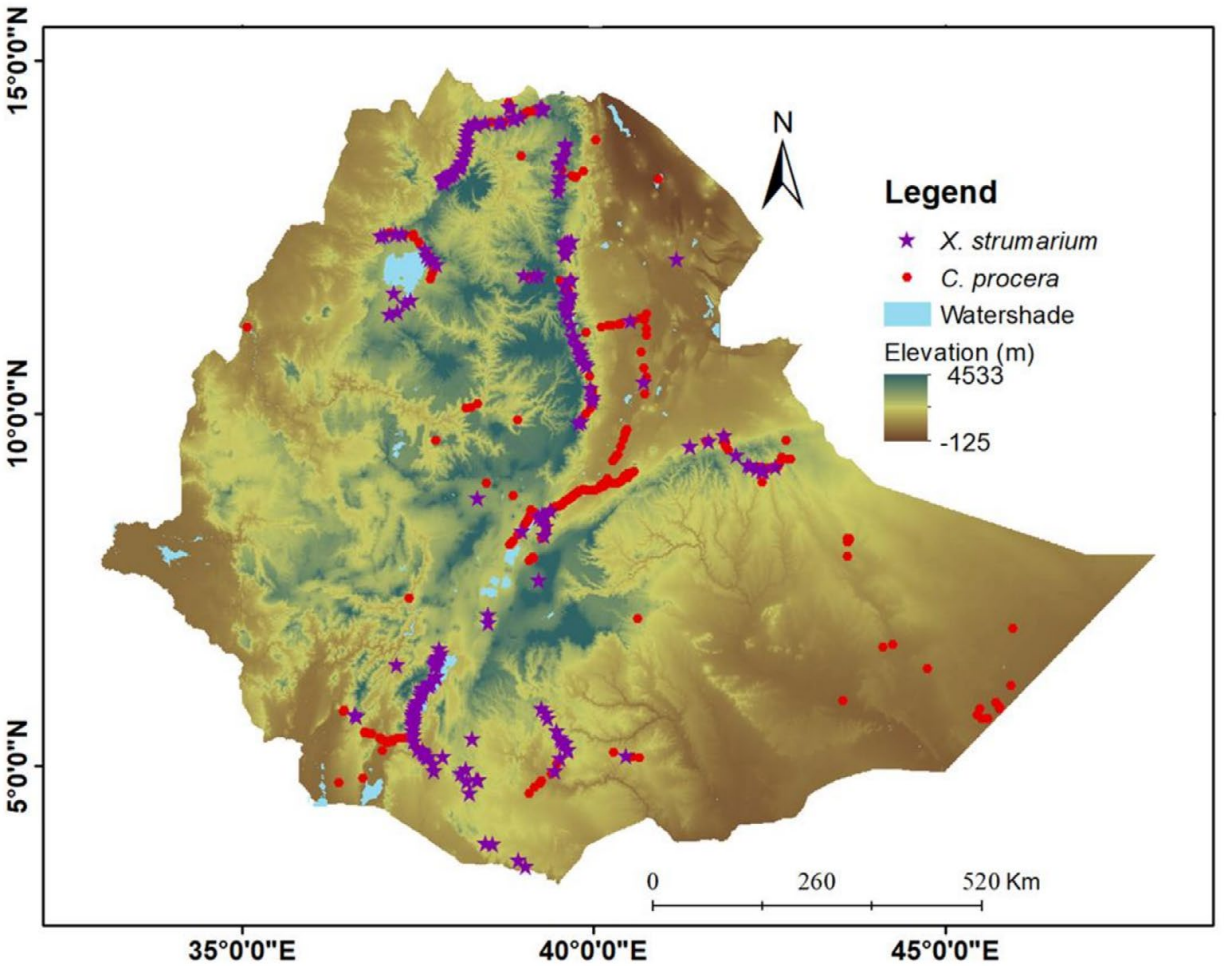
Topographic maps of Ethiopia showing the relief and geographic positions of occurrences of *C. procera* and *X. strumarium* across elevation ranges.

The present study was conducted in Ethiopia (Figure 2), using the *C. procera* and *X. strumarium* occurrence data. Voucher specimens of the invasive species (with collection numbers DMA001 for *C. procera* and DMA002 for *X. strumarium* ) were collected from study sites, pressed, and transported to the National Herbarium (ETH) for further identification. The samples were compared with the authenticated specimen in the National Herbarium, identified, and finally confirmed by experts in the field and deposited there for further reference. The species occurrence data used for this study were collected from a field survey, from the Global Biodiversity Information Facility (*n* = 179 for *C. procera* and *n* = 145 for *X. strumarium* ) (GBIF.org 2025a, 2025b). The GBIF data were carefully evaluated and cross‐checked with herbarium records. Records with accurate geographic coordinates were retained, whereas those with ambiguous locations were excluded. We used occurrence points of specimens collected from the 1980s onward since the accuracy of location data tends to diminish with specimen age (Bloom et al. 2018).

Ground field data were obtained from surveys conducted between October 2024 and February 2025 across various sites characterized by rich biodiversity and cultivated crop areas. These sites included the South Wollo Zone (Kalu and Tehuledere districts), areas along and within the Great Rift Valley, such as Adama to Chiro and Adama to Ziway, and parts of eastern Ethiopia, including the route from Dire Dawa to Jijiga and onward to the Ogaden region up to the Djibouti border. The field survey also extended to southern Ethiopia, covering areas from Arba Minch to Jinka and Mago National Park, as well as the stretch from Weyto to Omorate. During the field survey, *C. procera* was recorded at 154 locations and *X. strumarium* at 57 locations. Data were collected from farmland, grazing land, forests, highways, and roadsides, ensuring a minimum distance of 1 km between sampling sites.

A multi‐step data cleaning and rarefaction process was carried out to ensure the reliability of occurrence data. Occurrence points were spatially rarefied at a 1 km^2^ resolution using SDM Toolbox v2.6 (Brown 2014) to minimize spatial autocorrelation (Guisan et al. 2006). A total of 552 and 325 occurrence records were initially compiled for *C. procera* and *X. strumarium* , respectively, from field surveys, herbarium specimens, GBIF, and published literature (Erenso 2024; Amare et al. 2025).

Initially, 32 *C. procera* and 40 *X. strumarium* records were discarded because of missing coordinates or doubtful geographic information identified through visual inspection in ArcGIS and Google Earth. Subsequently, 4 and 5 records were excluded owing to taxonomic ambiguities or inconsistencies with known species distributions, verified against the *Flora of Ethiopia and Eritrea* (Hedberg et al. 1996; Tadesse 2004). Finally, 10 duplicate records for *C. procera* and 6 for *X. strumarium* were removed prior to spatial filtering. After cleaning, 506 and 274 verified and georeferenced records remained for *C. procera* and *X. strumarium* , respectively. Following spatial rarefaction, 330 and 202 unique occurrence points were retained for species distribution modeling (Tables S1 and S2).

### 2.2 Ecological Predictor Variables

Initially, we considered 23 environmental variables for modeling, including 19 bioclimatic factors (Bio1–Bio19), along with human footprint, altitude, aspect, and slope. The bioclimatic variables were obtained from the WorldClim 2.1 database (www.worldclim.org/bioclim) at a spatial resolution of 30 arc‐seconds (~1 km^2^), as this resolution provides a good balance between spatial detail and computational efficiency for ecological modeling (Bobrowski et al. 2021), on the basis of interpolated climate data from 1970 to 2000 (Fick and Hijmans 2017).

In addition to 19 bioclimatic variables, we included topographic and anthropogenic predictors to model habitat suitability. Elevation, aspect, and slope were derived from the Shuttle Radar Topography Mission digital elevation model (SRTM DEM; https://srt.csi.cgiar.org/). The slope angle map was specifically derived from the same digital elevation model. To represent anthropogenic disturbance, we used a Human Footprint Pressure raster (Venter et al. 2016) obtained from SEDAC (https://sedac.ciesin.columbia.edu/data/set/wildareas‐v3‐2009‐human‐footprint). This raster layer reflects the cumulative intensity of human activities such as roads, settlements, agricultural zones, and infrastructure on a scale from 0 to 50 with a spatial resolution of approximately 1 km^2^ (Di Marco et al. 2018; Campera et al. 2020; Hending et al. 2023).

All predictor layers were re‐projected, cropped, and resampled in R software version 4.3.3 to match the coordinate reference system, extent, and resolution of the bioclimatic variables. The coordinate reference system (CRS) of the current bioclimatic data was used as the reference. Elevation, aspect, slope, and human footprint pressure layers were projected to this CRS using the “project ()” function. Subsequently, these layers were cropped and resampled to match the spatial extent and resolution of the bioclimatic raster layers using “crop ()” and “resample ()” functions, respectively.

We stacked all environmental variables and extracted their values at species occurrence points as well as at 10,000 randomly generated background points. These background points captured the full range of environmental conditions across the study area, enabling more reliable variable selection and helping to reduce overfitting. To ensure variable independence, we retained only those variables with pairwise correlation coefficients below 0.7 and variance inflation factors (VIF) under 10 (Hair et al. 1986; Guisan et al. 2017). Variables were removed iteratively using a stepwise procedure on the basis of VIF, implemented with the “usdm” package in R (Naimi et al. 2014). This process retained 12 environmental variables for model development (Table 1).

**TABLE 1:** Selected variables and their Variance Inflation Factor (VIF).

No	Variables	Units	Code	VIF1^a^	VIF2^b^
1	Isorthemality (BIO2/BIO7) × 100	°C	BIO3	6.95	7.41
2	Temperature seasonality	°C	BIO4	7.45	7.90
3	Temperature annual range (BIO5‐BIO6)	°C	BIO7	2.17	1.99
4	Mean temperature of the driest quarter	°C	BIO9	3.44	3.15
5	Precipitation of the warmest month	mm	BIO13	6.73	2.79
6	Precipitation of the driest month	mm	BIO14	2.88	2.79
7	Precipitation seasonality	× 100	BIO15	3.08	2.94
8	Precipitation of the warmest quarter	mm	BIO18	2.38	2.30
9	Precipitation of the coldest quarter	mm	BIO19	3.56	3.41
10	Aspect	Degree		1.00	1.01
11	Slope	× 100		1.20	1.23
12	Human footprint	—		1.51	1.49

^a^ VIF1‐ Variance Inflation Factor of *C. procera*.

^b^ VIF2‐ Variance Inflation Factor of *X. strumarium*.

For future climate projections, the HadGEM3‐GC global circulation model (GCM) from the Coupled Model Intercomparison Project Phase 6 (CMIP6) (Michaelides 2023), developed by the UK Met Office Hadley Center, was used for future climate modeling. Two shared socioeconomic pathways (SSPs) were selected for projections targeting the years 2050 and 2070: SSP2‐4.5, representing intermediate greenhouse gas emissions with CO_2_ concentrations increasing until 2050 and then declining to net zero by 2100, and SSP5‐8.5, representing very high greenhouse gas emissions where CO_2_ levels triple by 2100 (Riahi et al. 2017). These scenarios were applied to assess the future potential suitable habitats of *C. procera* and *X. strumarium* .

### 2.3 Species Distribution Modeling

Species distribution prediction and projection were performed using an ensemble modeling approach that incorporates multiple algorithms, grouped into three main categories: profile‐based methods, classical regression techniques, and machine learning models (Hijmans and Elith 2019). In this study, we used three regression algorithms: Generalized Linear Models (GLM), Generalized Additive Models (GAM), and Multivariate Adaptive Regression Splines (MARS). We also applied four machine learning methods: Boosted Regression Trees (BRT), Maximum Entropy (MaxEnt), Random Forests (RF), and Support Vector Machines (SVM) to improve predictive accuracy and model robustness (Meynard and Quinn 2007; Santini et al. 2021). These models are extensively used in research and species conservation (Zhang and Li 2017) for their strong predictive accuracy (Elith et al. 2008; Wang et al. 2011; Kaky et al. 2020).

The selection of modeling algorithms was guided by their compatibility with the characteristics of the dataset and their well‐documented performance in ecological applications (Hastie et al. 2009; Merow et al. 2014). To address potential sampling bias and prevalence issues, we applied case weights to the presence and pseudo‐absence data during model calibration (Chala et al. 2016). Following algorithm selection, we optimized model performance by evaluating both default and customized parameter settings. This process included adjustments related to background point generation, number of iterations, learning rate, feature types, and the number of trees. Species distribution models were implemented using the sdm package in R (Naimi and Araújo 2016), which facilitates ensemble modeling across multiple algorithms. Models were trained using 70% of the occurrence dataset and evaluated with the remaining 30% through a 10‐fold subsampling approach (Wang et al. 2022).

Algorithm‐specific configurations were applied to enhance model reliability. For Boosted Regression Trees (BRT), we set the learning rate to 0.05, used 500 trees, and applied a bag fraction of 0.75. Random Forest (RF) was configured with 500 trees. Generalized Linear Models (GLM) and Generalized Additive Models (GAM) were specified using a binomial family (Hastie et al. 2009). The MaxEnt model was run with 10,000 iterations and a regularization multiplier of 1 (Ahmed, Chala, et al. 2023; Kufa et al. 2025). For Support Vector Machines (SVM), a radial kernel with a cost parameter of 1 was applied, and the Multivariate Adaptive Regression Splines (MARS) model was set with degree equal to 2 and nprune equal to 30.

To provide absence data, 10,000 background points were randomly sampled across the study area using the gRandom method within the sdmData function. These background points were used as pseudo‐absences for algorithms requiring binary data and as background for MaxEnt, ensuring consistency across modeling approaches. Final ensemble predictions were generated by averaging outputs from all models, weighted by their respective True Skill Statistic (TSS), in line with established best practices to improve accuracy and reduce predictive uncertainty (Dormann et al. 2018).

### 2.4 Model Validation and Mapping

The performance of the model was assessed using both threshold‐independent and threshold‐dependent evaluation metrics. Receiver operating characteristic (ROC) curves (Figures S1 and S2) were generated, and the area under the curve (AUC) was calculated to provide a threshold‐independent measure of model accuracy (Allouche et al. 2006). AUC values range from 0 to 1, where 0.5 indicates a performance no better than random, values between 0.7 and 0.8 are considered fair, 0.8 to 0.9 indicates good performance, and scores above 0.9 reflect excellent discriminatory power. Threshold‐dependent metrics, including true skill statistic (TSS), sensitivity, and specificity, were also used. TSS values range from −1 to +1, with 1 indicating perfect prediction, 0 representing random performance, and values above 0.6 generally reflecting good model performance. Negative TSS values suggest that the model performs worse than random (Zou et al. 2011; Bahn and McGill 2013).

To assess whether there were significant differences in model performance among the algorithms, we compared the area under the curve (AUC) scores obtained from 10 replicate runs for each algorithm. The Kruskal–Wallis test was applied to evaluate overall differences among algorithms. When a significant difference was detected, pairwise comparisons between algorithms were conducted using the Wilcoxon rank‐sum test (also known as the Mann–Whitney *U* test). All statistical analyses were performed in R (R software version 4.3.3), and *p*‐values < 0.05 were considered statistically significant.

Ensemble models were used to predict the suitable habitats and distribution ranges of *C. procera* and *X. strumarium* under current climate conditions (baseline) for assessing potential impacts of future climate change. Given the continuous nature of the model outputs, an optimal threshold for species presence was determined by maximizing the sum of sensitivity and specificity (max (se + sp)). This method ensures a balanced trade‐off between omission and commission errors and corresponds with the maximization of the True Skill Statistic (TSS), a widely used metric for evaluating model accuracy in ecological studies (Liu et al. 2005; Allouche et al. 2006; Jimenez‐Valverde and Lobo 2007).

To further improve the robustness of the predictions, a weighted mean threshold was calculated using TSS values rather than relying on a simple average, following the approach of Liu et al. (2013). On the basis of this threshold, pixels were classified as suitable or unsuitable in the final binary habitat maps. The distributions of *C. procera* and *X. strumarium* across Ethiopia were then mapped using ArcGIS 10.8. Future projections were also generated for the years 2050 and 2070 under two shared socioeconomic pathways: SSP2‐4.5 (intermediate) and SSP5‐8.5 (high emissions). ArcGIS 10.8 was used to quantify the spatial extent of areas classified as unsuitable, suitable, lost, gained, or unchanged (remained suitable or unsuitable). These outputs were used to evaluate range shifts and assess the potential impacts of climate change on the future distribution of both species, including patterns of habitat gain, loss, and redistribution (Fordham et al. 2012).

## 3 Results

### 3.1 Model Performance and Variable Importance

All individual algorithms and their overall ensemble modeling results showed high predictive power and consistent, strong performance across the evaluation metrics of AUC, TSS, Sensitivity, and Specificity for *C. procera* and *X. strumarium* . The average AUC values were 0.93 ± 0.02 for *C. procera* and 0.94 ± 0.02 for *X. strumarium* , whereas the corresponding TSS values were 0.74 ± 0.05 and 0.79 ± 0.06, respectively. Among the predictive models, the machine learning algorithms Random Forest (RF) and MaxEnt showed the highest performance of AUC and TSS metrics for both species (Table 2).

**TABLE 2:** Performance evaluation of each SDM using different statistical parameters.

Species	Evaluation metrics	Models								
		BRT	RF	GLM	GAM	MaxEnt	SVM	MARS	SD	Ensemble
*C. procera*	AUC	0.91	0.95	0.92	0.94	0.94	0.89	0.93	0.02	0.93
	TSS	0.68	0.81	0.71	0.77	0.77	0.67	0.74	0.05	0.74
	Sensitivity	0.78	0.90	0.81	0.86	0.85	0.73	0.84	0.05	0.82
	Specificity	0.90	0.91	0.90	0.91	0.93	0.94	0.90	0.01	0.91
*X. strumarium*	AUC	0.90	0.97	0.93	0.96	0.97	0.93	0.95	0.02	0.94
	TSS	0.69	0.86	0.74	0.83	0.85	0.76	0.80	0.06	0.79
	Sensitivity	0.80	0.93	0.90	0.93	0.95	0.85	0.92	0.05	0.90
	Specificity	0.89	0.93	0.85	0.91	0.90	0.93	0.89	0.03	0.90

Abbreviation: SD, standard deviation.

**FIGURE 3:**
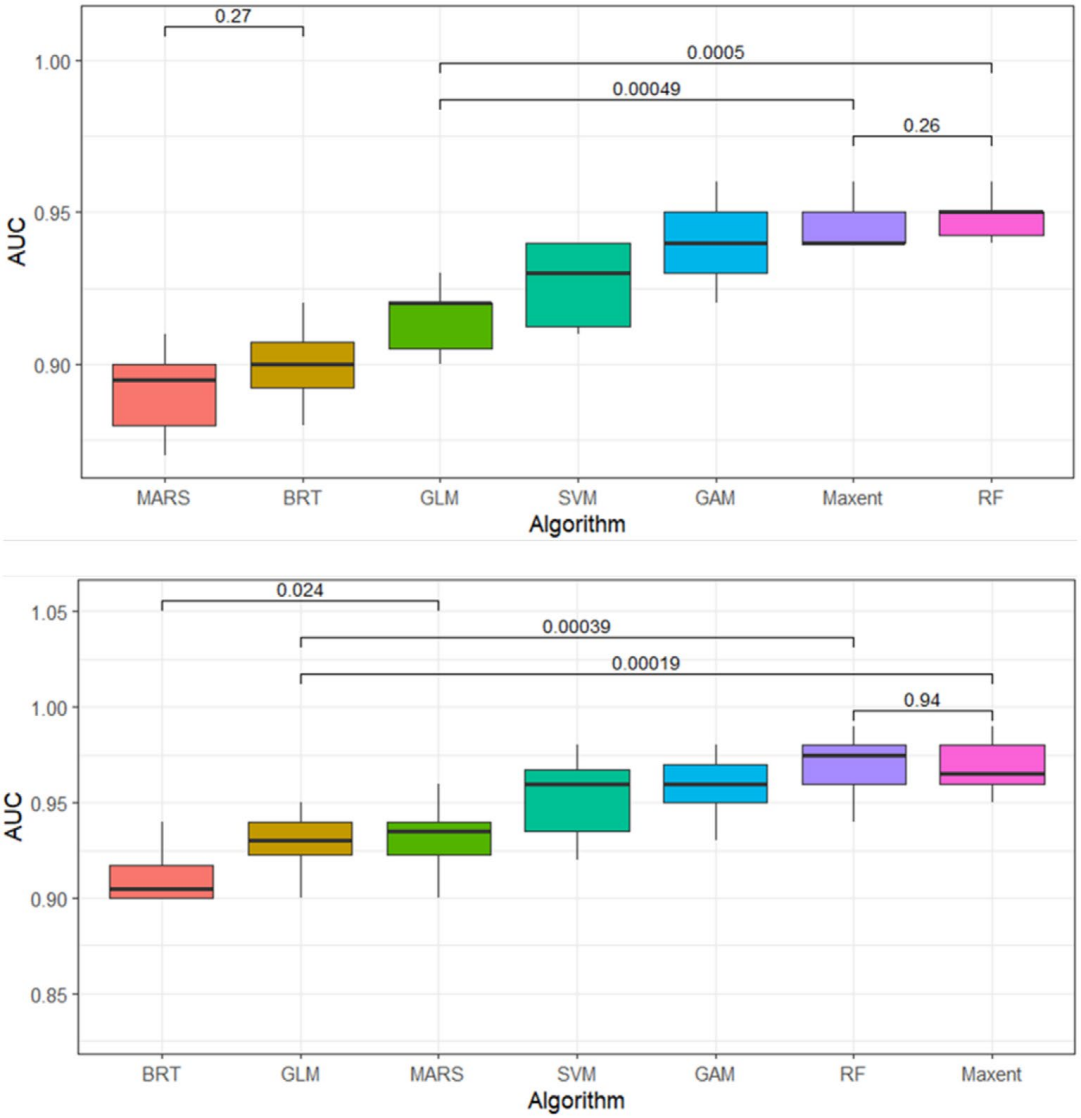
Boxplots show the model evaluation performance of AUC scores across 10 replications for each algorithm for *C. procera* and *X. strumarium* , respectively, from top to bottom. Pairwise comparisons were conducted using the Wilcoxon rank‐sum test. Statistically significant differences are indicated, with corresponding *p*‐values displayed on the plot.

Significant differences were observed in the algorithm performance. Machine learning models such as RF and MaxEnt significantly differ from regression‐based models like GLM and GAM (*p* < 0.05) (Figure 3). However, the AUC and TSS values remained consistent between training and test datasets, indicating stable model performance. The standard deviations of the evaluation metrics, which ranged from 0.01 to 0.06 and from 0.01 to 0.05 for *C. procera* and from 0.02 to 0.06 for *X. strumarium* , suggest minimal variability, demonstrating the data quality for predictions for the ability of the predictions to identify the high‐potential areas as providing suitable habitat for the targeted species. Overall, predictive outputs remained stable across different model settings, repeated runs, and datasets, highlighting the robustness of the results (Table 2).

**FIGURE 4:**
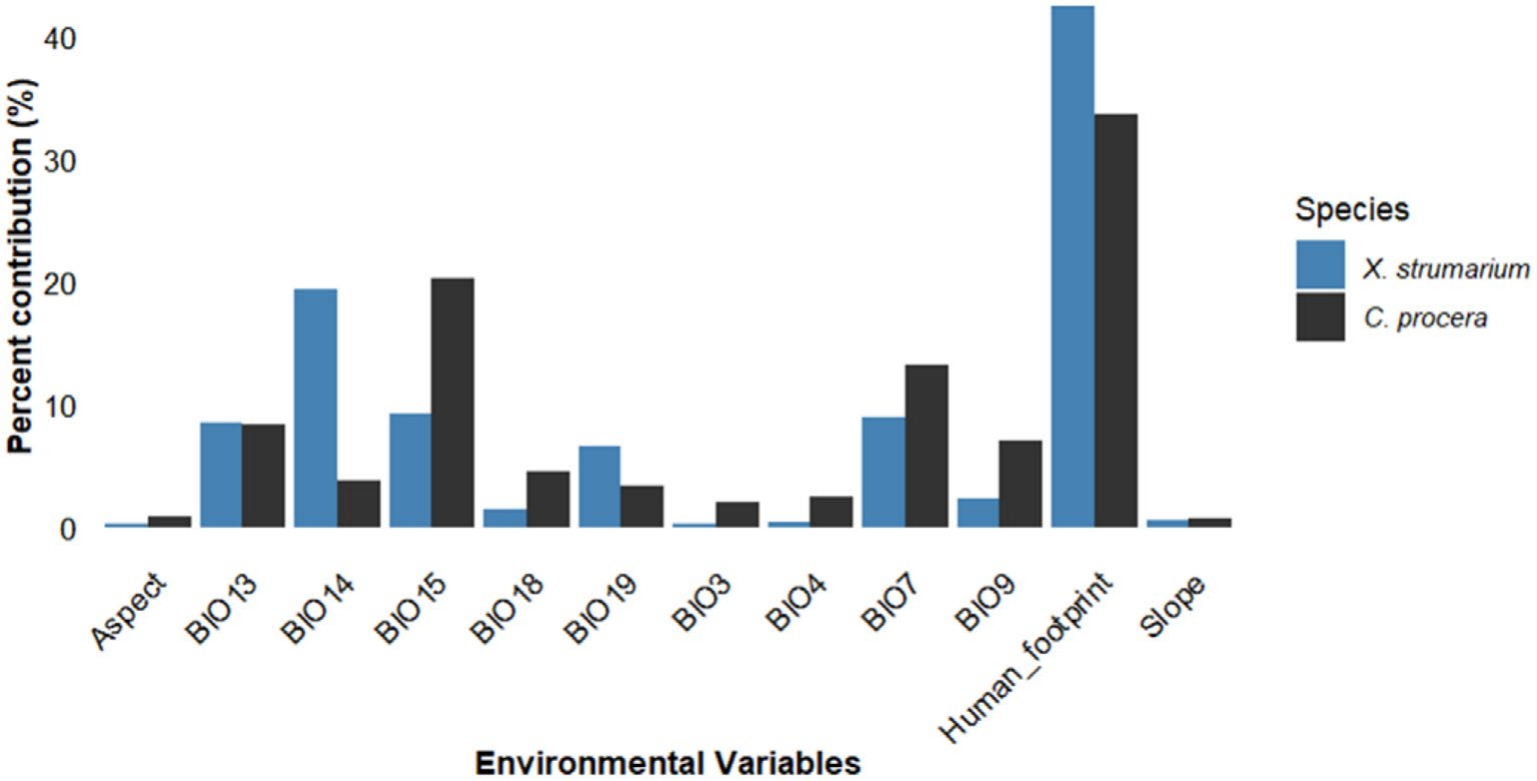
Bar graph showing the contributions of the predictor variables for ensemble algorithms for predicting habitat suitability for *C. procera* and *X. strumarium* . Values represent the averaged relative importance derived from seven algorithms used in the ensemble.

The overall results showed that the human footprint was a notably influential factor affecting the distribution and suitable habitats of *C. procera* (33.53%) and *X. strumarium* (42.41%). This is followed by precipitation seasonality (20.28%) and temperature annual range (13.25%) for *C. procera* and precipitation of the driest month (19.32%) and precipitation seasonality (9.14%) for *X. strumarium* (Figure 4).

### 3.2 Habitat Suitability Modeling

**FIGURE 5:**
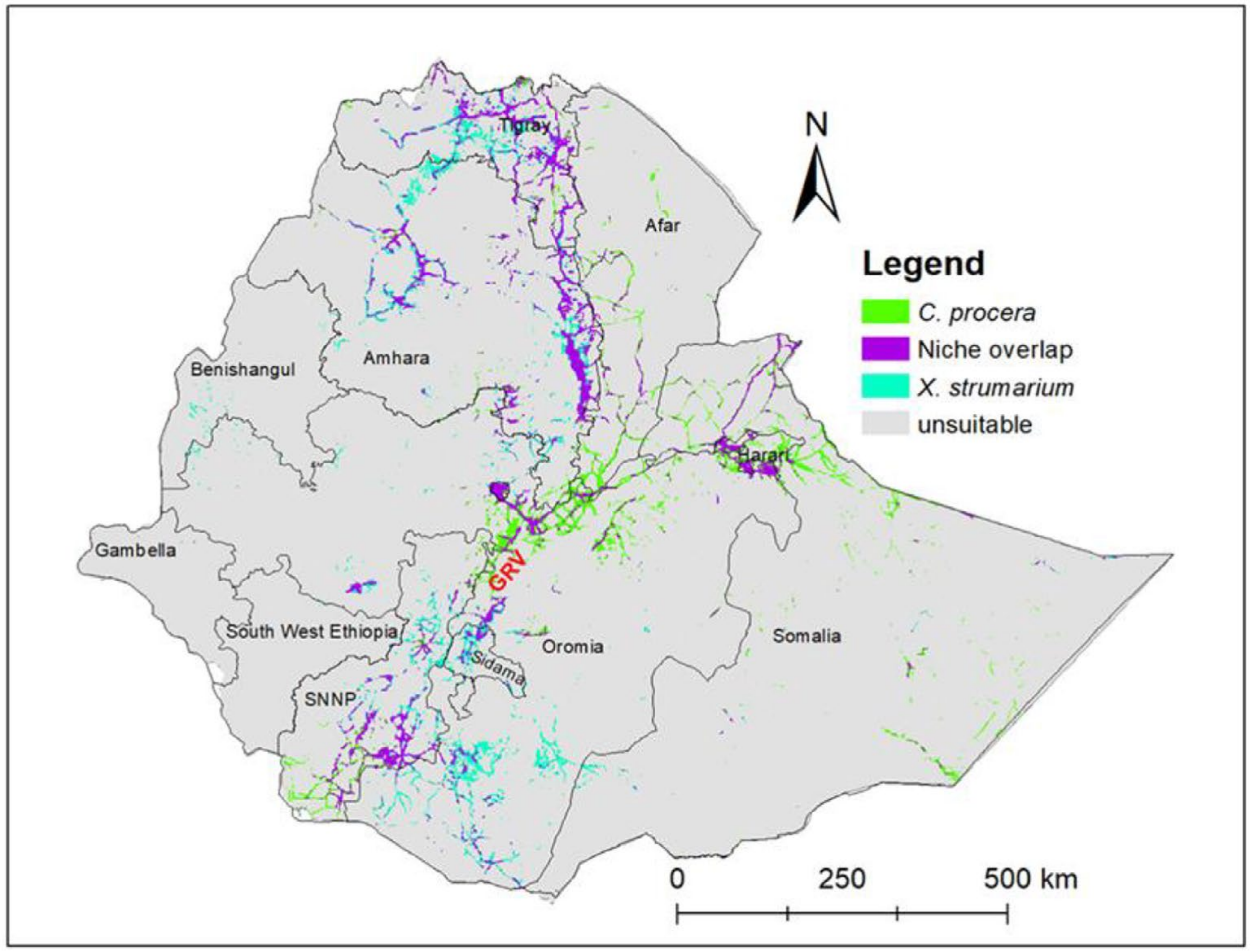
Suitable habitat distribution for *C. procera* and *X. strumarium* under current climatic conditions in different regions of Ethiopia, including areas of overlap (violet), areas suitable only for *C. procera* (green), areas suitable only for *X. strumarium* (turquoise), and areas unsuitable for both species (gray). GRV, The Great Rift Valley.

Under the current climate, predicted maps showed that suitable habitats for *C. procera* are predominantly found in the northern, central, southern, and eastern lowlands of Ethiopia. The predicted suitable habitats were more extensive in Dire Dawa, Harari, Somali, Afar, Oromia, Tigray, Amhara, Southern Ethiopia, and to some extent the Gambella region. The lowlands of Dire Dawa, Harari, Somali, and Afar are particularly vulnerable, as they are connected by highways to Djibouti, which may facilitate the spread of the species (Figure 5).

Likewise, *X. strumarium* has currently invaded northern Ethiopia, mainly in the Tigray and Amhara regions, as well as central Ethiopia, including parts of Oromia, southern Ethiopia within the Southern Nations, Nationalities, and Peoples region, and eastern Ethiopia in Dire Dawa and Harari. It has also been observed to some extent in the Benishangul Gumuz and Somali regions (Figure 5).

**FIGURE 6:**
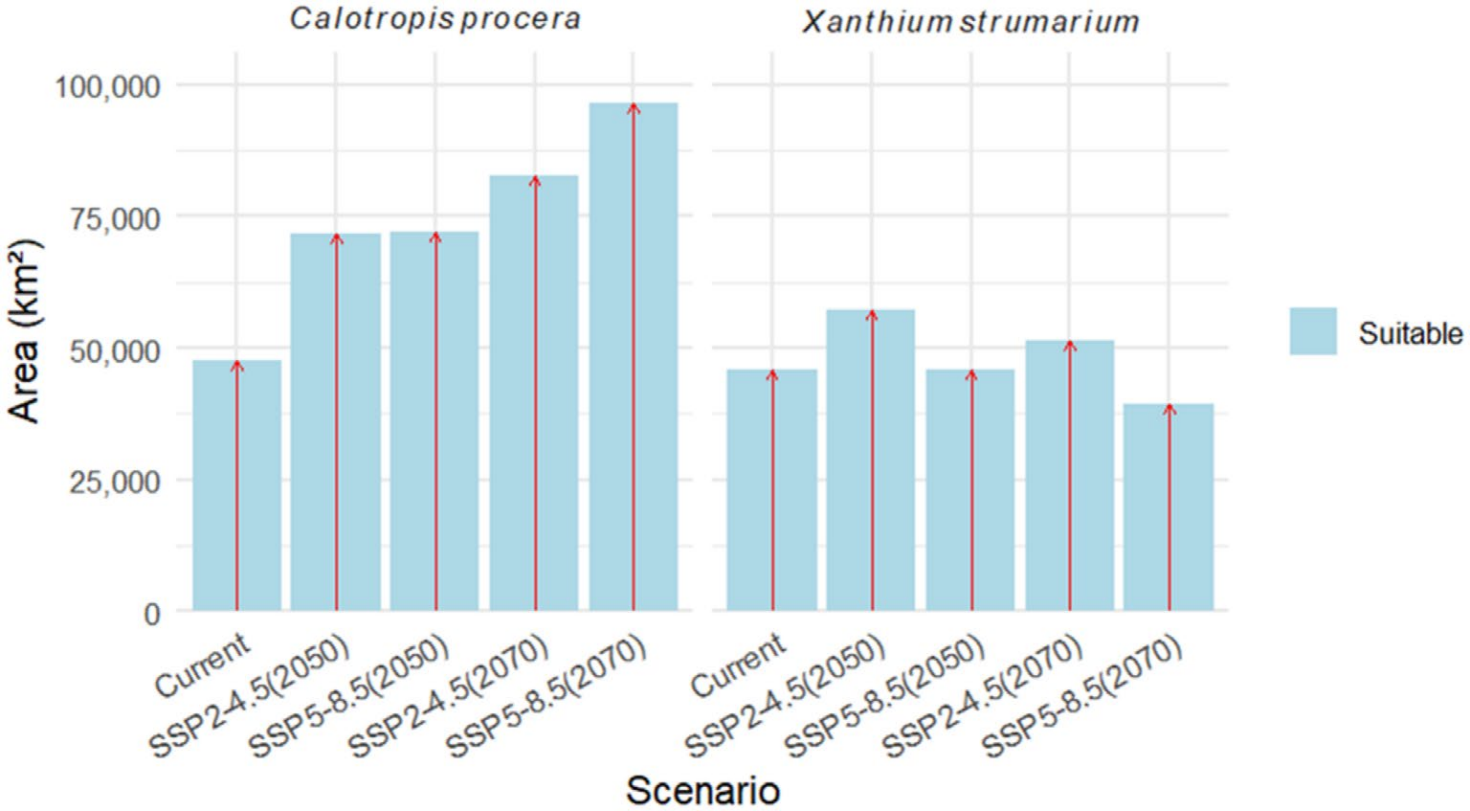
Area of suitable habitats predicted under current and future climate scenarios for both invasive weed species.

Model projections indicate that the current suitable habitat for *C. procera* and *X. strumarium* covers approximately 47,652.37 km^2^ (4.2%) and 45,900.76 km^2^ (4.05%), respectively (Figure 6). The maximum niche overlap between the two invasive species is estimated at 27,062 km^2^, suggesting areas of potential co‐establishment and competitive interaction (Figure 5).

### 3.3 Future Projection

**FIGURE 7:**
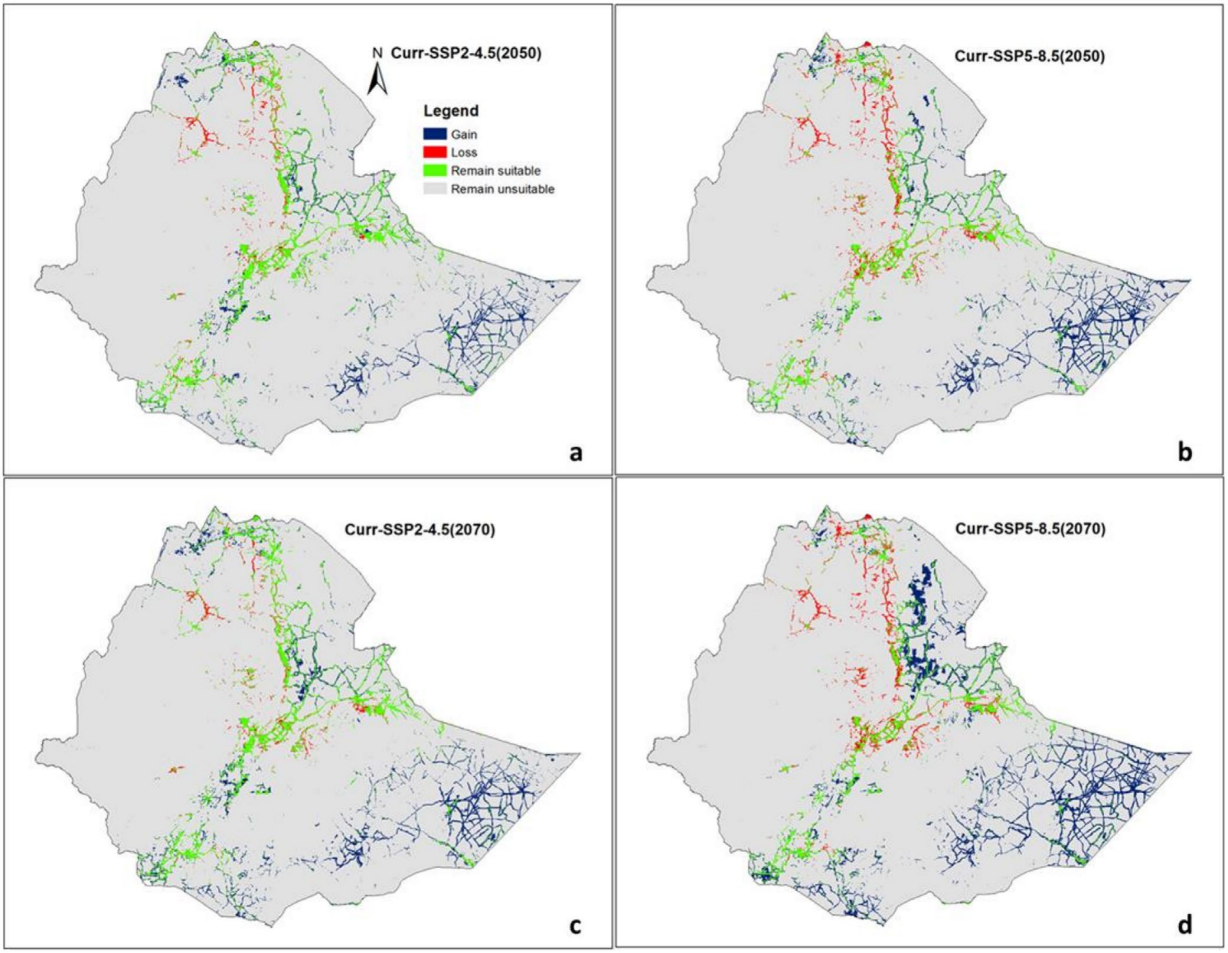
Projected habitat suitability area changes for *C. procera* under Current–2050s (a: Medium scenario, b: Worst scenario) and Current–2070s (c: Medium scenario, d: Worst scenario) conditions.

**FIGURE 8:**
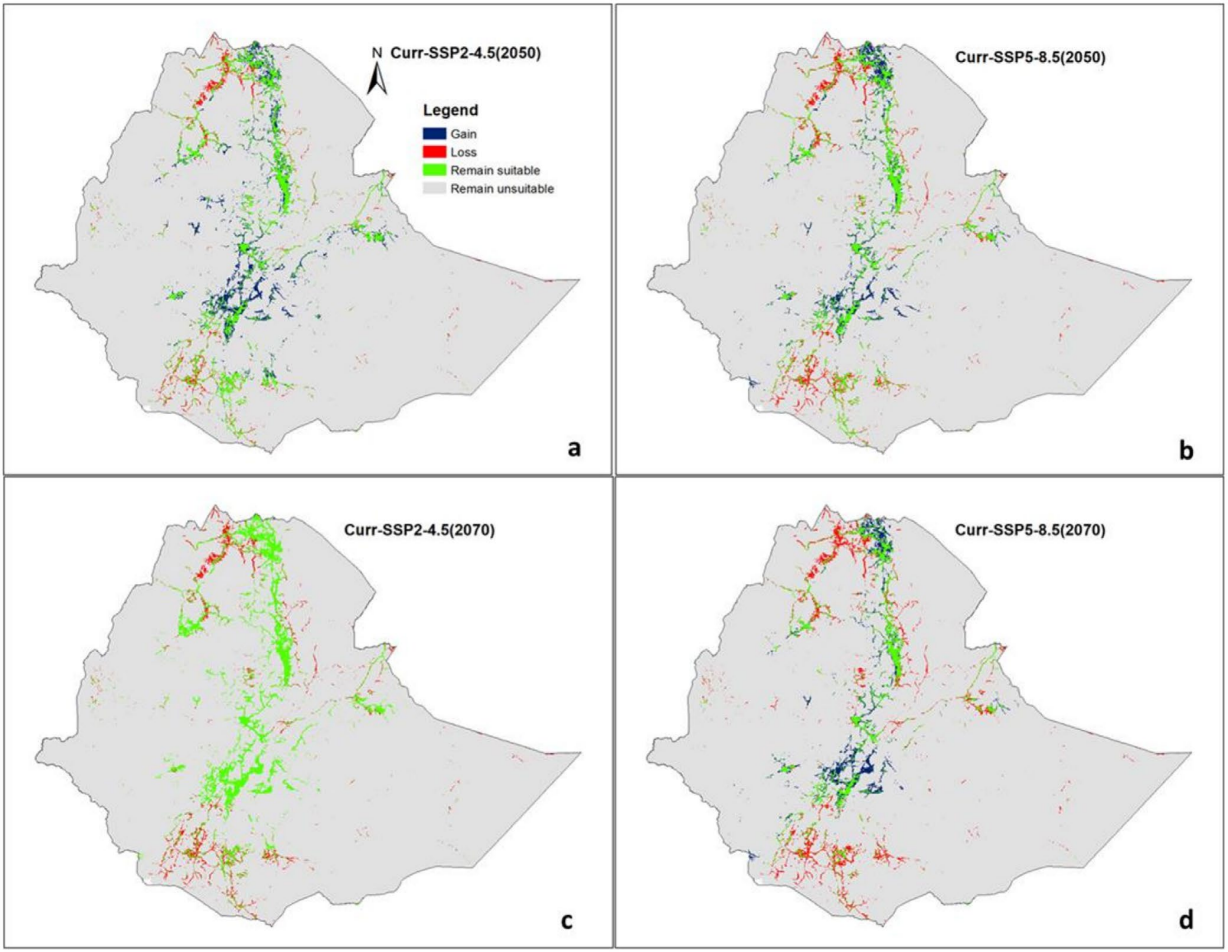
Projected habitat suitability area changes for *X. strumarium* under Current–2050s (a: Medium scenario, b: Worst scenario) and Current–2070s (c: Medium scenario, d: Worst scenario) conditions.

We projected the potential and changes in the distribution of suitability ranges for *C. procera* and *X. strumarium* under two future emission scenarios: a moderate (SSP2–4.5) and the worst (SSP5–8.5). Compared to the current (baseline), *C. procera* is projected to gain suitable habitats under both moderate and worst climate scenarios, whereas *X. strumarium* is expected to gain suitable habitats under the moderate scenario (Figures 7 and 8, Figures S3 and S4).

The model predicts an expansion of suitable habitat for *C. procera* from 47,652.37 km^2^ to 96,629.26 km^2^ (Figure 6), with a gain of 65,461.41 km^2^ (137.43%) under the worst scenario (2070s) (Table 3, Figure 7). For *X. strumarium* , suitable habitat expands under the moderate scenario, increasing from 45,900 km^2^ to 57,120.73 km^2^ by the 2050s (Figure 6). However, under the worst scenario, the species shows a reduction in suitable habitat to 39,300 km^2^, with a net loss of 6588.97 km^2^ (14.36%) (Figures 6 and 8, Table 3).

**TABLE 3:** Temporal changes in the projected range size of *C. procera* and *X. strumarium* under different climate scenarios in Ethiopia.

Species	Scenarios	Loss km^2^	Remain suitable km^2^	Gain km^2^	% Loss	% Gain	Species range change (%)	Current range size km^2^	Future range size km^2^
*C. procera*	SSP2‐4.5 (2050)	8272.68	39,366.55	32,405.26	17.37	68.02	50.66	47,639.24	71,771.81
	SSP5–8.5 (2050)	15,022.53	32,612.14	39,528.75	31.54	121.21	89.67	47,634.66	72,140.89
	SSP2‐4.5 (2070)	7421.88	40,213.64	42,585.25	15.58	89.40	73.82	47,635.51	82,798.89
	SSP5‐8.5 (2070)	16,526.53	31,105.83	65,461.41	34.70	137.43	102.73	47,632.36	113,093.76
*X. strumarium*	SSP2‐4.5 (2050)	10,270.04	35,620.85	21,494.15	22.38	46.84	24.46	45,890.89	57,115.00
	SSP5–8.5 (2050)	15,880.42	30,009.81	15,839.57	34.61	34.52	0.09	45,890.24	45,849.38
	SSP2‐4.5 (2070)	14,889.52	31,000.12	20,465.19	32.45	44.60	12.15	45,889.65	51,465.32
	SSP5‐8.5 (2070)	22,114.73	23,770.70	15,525.76	48.20	33.84	14.36	45,885.43	39,296.46

Under both current and future climate conditions, highly suitable habitats for both species were observed extending from Northern Ethiopia (Tigray) through Central Ethiopia, the Great Rift Valley, Southern and Eastern regions, including Dire Dawa and the Somali region, with *C. procera* projected to expand further across the entire Somali region up to the borders of Somaliland (Figure 7, Figure S3).

## 4 Discussion

This study assesses the impacts of climate change and environmental predictors on the predicted suitable habitats for invasive species in Ethiopia over time, providing valuable insights to guide management efforts and minimize their potential ecological and economic impacts. Our results demonstrate that strong model performance across all averaged evaluation metrics enhances the accuracy of identifying high‐potential suitable habitat for a possible management plan. In this study, we applied different algorithms in different data settings, default, iterations, replication, learning rate, features, number of trees, and complexity level. The highest performance and consistent results are more reliable for discriminating suitable and unsuitable habitats (Dorji et al. 2022; Melese, Woldu, et al. 2025). These consistent results across the evaluation metrics highlighted data quality (Ahmed, Bekele, et al. 2023), which strengthened the reliability of current predictions for *C. procera* and *X. strumarium* .

### 4.1 Major Environmental Determinants of the Potential Distribution of Invasive Species

In the present study, under all algorithms and settings, the results showed that the human footprint is the most influential predictor factor determining the distribution and habitat suitability for both *C. procera* and *X. strumarium* . This underscores its central role in facilitating the spread of these invasive species. In a comparable manner, the expansion and distribution of invasive species such as *C. procera* are influenced more by anthropogenic pressures than by bioclimatic attributes (Mbambala et al. 2021). The human footprint, which reflects the intensity of human activities such as transportation infrastructure, trade routes, and tourism development, more likely creates disturbed habitats and corridors that promote the spatial expansion of these invasive weeds (Leu et al. 2008; Gallardo et al. 2015; Wan et al. 2017). Additionally, the human footprint has a strong positive correlation with their species diversity and richness (Wang and Xu 2016).

Bioclimatic variables also play a significant role in determining the potential distribution of *C. procera* and *X. strumarium* . Temperature and precipitation‐related variables, in particular temperature annual range, precipitation of the driest month, and precipitation seasonality, were found to be important in shaping habitat suitability (Mbambala et al. 2021). These climatic factors influence seed germination, growth rates, flowering duration, seed quality and quantity, and overall survival by affecting temperature extremes and water availability during critical growth periods. Such ecological sensitivities contribute to the successful establishment and expansion of these invasive species across environmentally suitable areas (Turbelin and Catford 2021).

For *C. procera* , the temperature annual range and precipitation seasonality were identified as key bioclimatic variables influencing its potential distribution, suggesting that the species is particularly responsive to environments with high temperature variability and irregular rainfall patterns (Iqbal et al. 2022). This ecological preference aligns with its known physiological traits, such as tolerance to heat stress, deep rooting capacity, and water‐use efficiency, which allow it to thrive under fluctuating temperatures and seasonal drought. These adaptations enable *C. procera* to establish and spread widely in arid and semi‐arid regions where such climatic conditions prevail (Hassan et al. 2015).

*X. strumarium* was primarily influenced by the annual temperature range, precipitation of the driest month and precipitation seasonality, indicating that both temperature variability and precipitation dynamics play significant roles in determining its suitable habitat. These results align with previous experimental studies showing that the species responds strongly to medium to high temperatures with daily thermal fluctuations, which enhance germination and seedling development. Together, these findings suggest that precipitation governs the broader spatial distribution of *X. strumarium* , whereas temperature, both seasonal and short‐term, contributes to its successful establishment and early growth at local scales (Lechowicz 1984; Norsworthy and Oliveira 2007).

### 4.2 Potential Invasive Range of *C. procera* and *X. strumarium*

The climate suitability analysis for the period 1970 to 2000 indicates that approximately 4.2% of Ethiopia's total area was favorable for the establishment of *C. procera* . Specifically, the Great Rift Valley along and in it, as well as the northern, eastern, central, southern, and southeastern regions, were identified as hotspots for the climatically suitable distribution of *C. procera* . This finding aligns with the natural range of *C. procera* , which is typically associated with arid and semi‐arid environments, particularly those found in the Great Rift Valley and surrounding lowland areas (Hedberg et al. 1996). The species is well adapted to dry climates and disturbed habitats, allowing it to thrive in these regions. The invasion tends to intensify along major transportation corridors, particularly the highways from Adama to Djibouti, Hawassa, Arba Minch, and the roads leading to Wollo and Tigray. This pattern highlights the role of the human footprint, including vehicle movement, in facilitating seed dispersal and the spread of this invasive species (Melese, Woldu, et al. 2025). Recently, because of increasing anthropogenic pressures, many plant species have begun migrating from lowland to highland areas (Jump et al. 2012). Some of these species show a high potential for adaptation in the Ethiopian highlands, where several flagship species are found. This is evident in the case of *Lobelia rhynchopetalum*, which has been pushed into the sky islands of Ethiopia because of anthropogenic pressures and climate change (Chala et al. 2016).

Similarly, *X. strumarium* primarily occupied the low‐ to mid‐altitude highland parts of Ethiopia, with central, northern, and southern regions being the most affected. It is mainly found in the Shewa, Wollo, Tigray, Sidama, and Gamo Gofa floristic regions, which aligns with a previous study (Tadesse 2004). In contrast to *C. procera* , which shows broader climatic suitability across arid and semi‐arid regions, including the Somalia floristic region, the suitable habitats for *X. strumarium* were generally limited and decreased in the Somalia floristic region.

Another concerning factor that aggravates the impact of these invasive weeds is their overlapping distribution across multiple regions, including northern, central, and southern Ethiopia. These regions are rich in both biodiversity and crop production, resulting in a compounded threat to ecologically and economically valuable areas (Brandt et al. 2023). Several biodiversity‐rich zones, including Nech Sar National Park and Maze National Park, which serve as reservoirs for diverse flora and fauna, are also affected. Although both species contribute to this overlapping invasion to some extent, *C. procera* is particularly widespread in the Afar and Ogaden areas, including Awash National Park.

Future climate change is likely to facilitate the expansion of suitable habitats for *C. procera* and *X. strumarium* , consistent with previous findings on the projected spread of aggressively expanding species under changing climatic conditions (Pyke et al. 2008; Finch et al. 2022; Melese, Woldu, et al. 2025). Compared to the current extent, model projections indicate that the area suitable for *C. procera* may expand under both future climate scenarios. Under the worst‐case scenario, the suitable habitat is projected to increase substantially, suggesting a potential range expansion in response to future climatic conditions. Habitat gain is primarily concentrated in the eastern, northeastern, southern, and southeastern parts of the country, particularly in the lowlands of the Somali and Afar regions. Native to Africa and Asia, *C. procera* demonstrates a remarkable ability to persist and remain productive under extremely arid conditions (Kaur et al. 2021). However, some habitat loss is projected in northern Ethiopia, likely because of human activities. In areas with intensive agricultural activity, invasive species management may be more active, reflecting a higher human footprint characterized by land use, cultivation, and direct intervention in the landscape (Leu et al. 2008).

On the contrary, model projections indicate that the invasive weed *X. strumarium* , originally native to the Americas, could potentially expand its suitable habitat across various parts of the country, particularly under the medium emission scenario. The species is projected to extend its distribution into the central and northern regions. However, unlike *C. procera* , *X. strumarium* does not tolerate arid and semi‐arid conditions as effectively. Its distribution is instead associated with regions that have moderate temperatures, which are more conducive to its reproductive success (Lechowicz 1984; Norsworthy and Oliveira 2007).

The current prediction of invasive species' distribution and habitat suitability provides a critical baseline for guiding future control and management efforts by identifying areas at greatest risk (Crossman et al. 2011; Chai et al. 2016). The two invasive species, *Calotropis procera* and *Xanthium strumarium* , pose significant threats to native ecosystems, agricultural productivity, and biodiversity. *C. procera* reduces maize and wheat yields by decreasing leaf area, plant height, and biomass through allelopathic effects (Umar et al. 2014). This species is spreading from roadsides into various land use types, particularly impacting agricultural and grazing lands, thereby challenging smallholder farmers in the East Shewa Zone of Oromia, Ethiopia (Erenso 2024). Similarly, *X. strumarium* adversely affects crops such as *Guizotia abyssinica* , *Linum usitatissimum* , and *Sorghum bicolor* through allelopathic interference, leading to reduced growth and yield (Seifu et al. 2024; Amare et al. 2025). In sorghum fields, the presence of *X. strumarium* has been shown to reduce aboveground dry matter and leaf area index (LAI), with the degree of reduction depending on weed density. Yield losses reached as high as 79.2% and 93.1% at maximum *X. strumarium* density in Haramaya and Babile, respectively (Amare et al. 2025). Moreover, the suitable habitat of *X. strumarium* overlaps with key areas where the important crop *Eragrostis tef* is cultivated, posing an additional threat to national food security (Melese, Asfaw, et al. 2025).

Both species exhibit strong invasive potential driven by a combination of physiological and ecological traits. Their high phenotypic plasticity allows them to tolerate broad ranges of moisture, temperature, and soil conditions, whereas rapid growth, early maturation, and prolific seed production enhance colonization success. The approximately 40% overlap in suitable habitat suggests similar ecological requirements, including tolerance to disturbance and wide climatic adaptability. Nevertheless, resource partitioning through differences in phenology or microhabitat preferences may reduce direct competition. In areas of co‐occurrence, competitive exclusion may still occur, with the more stress‐tolerant or faster‐growing species prevailing. Understanding these interactions is crucial for predicting invasion dynamics, particularly under climate change scenarios that may expand their overlapping ranges.

Mapping invasive species is essential for identifying high‐risk areas and guiding early warning and management strategies. By overlaying predicted suitable habitats with land‐use types such as forests, grazing lands, and agricultural areas, the model highlights priority zones for intervention and reveals key environmental drivers of invasion. In this study, the human footprint emerged as a major factor promoting expansion, underscoring the importance of regulating land use and limiting seed dispersal, especially in areas identified as highly suitable for invasion.

### 4.3 Limitations of the Study: Future Research Directions

Although ensemble modeling helped lower prediction uncertainty, the use of a single global circulation model (GCM) constrained the ability to reflect the full spectrum of future climate conditions. Invasive species are also often not in climatic equilibrium with their environment, and because they have not yet occupied all suitable habitats, projections of their potential spread beyond current ranges remain uncertain. These uncertainties suggest that, although the study effectively identifies environmental factors influencing current distributions, it may underestimate their potential future expansion. Moreover, because the analysis was mainly based on climatic and environmental variables, it did not explicitly consider biotic interactions such as competition or dispersal constraints. In future work, it would be better to incorporate multiple climate models, field validation, and experimental assessments of physiological tolerances to improve prediction accuracy. Integrating ecological, physiological, and genetic information would also provide a more comprehensive understanding of invasion dynamics under changing environmental conditions.

## 5 Conclusions

Our distribution and habitat suitability modeling for two ecologically and economically impactful invasive species, *C. procera* and *X. strumarium* , in Ethiopia used an ensemble modeling approach. The study identified the human footprint as the primary factor influencing their distribution, followed by bioclimatic attributes. An ensemble distribution model demonstrates that wide ranges across the country are covered with the potential suitable habitats of these invasive species under current and future scenarios, which underscores the areas and the indigenous plant species that are vulnerable to challenges from these invasive species. These invasive species have spread across the northern, central, southern, and eastern lowland regions of Ethiopia, particularly in arid and semi‐arid areas. The current and future habitat suitability maps reveal considerable overlap with the country's agriculturally productive and biodiversity‐rich areas, and their invasiveness is further amplified by a 40% overlap in suitable habitats, posing serious ecological and economic threats. Over time, their expansion is projected to be drastic, and their impacts increasingly severe, underscoring the need for urgent and effective management interventions. Therefore, targeted removal along roadsides, highways, and river channels, coupled with organized community‐based weeding campaigns across various land‐use types, can play a critical role in curbing their spread and mitigating their ecological impacts, especially those driven by human activity. Following de‐weeding, replanting native or non‐invasive vegetation can help restore ecological balance and suppress weed re‐establishment. Furthermore, citizen science initiatives, supported by agricultural experts and extension workers, can strengthen community participation in monitoring, reporting, and managing infestations, while also promoting awareness creation about invasive species and their ecological and economic impacts. Such collaborations can enhance early detection, promote knowledge sharing, and ensure long‐term stewardship of reclaimed landscapes.

## Author Contributions

**Daniel Melese:** conceptualization (equal), data curation (equal), formal analysis (lead), methodology (lead), resources (lead), software (lead), validation (equal), writing – original draft (lead). **Mulatu Ayenew Aligaz:** formal analysis (equal), validation (equal), writing – review and editing (equal). **Ahmed Seid Ahmed:** conceptualization (equal), data curation (equal), methodology (supporting), validation (equal), writing – review and editing (equal).

## Conflicts of Interest

The authors declare no conflicts of interest.

## Supporting information


**Appendix S1:** ece372577‐sup‐0001‐AppendixS1.docx.

## Data Availability

The data supporting the findings of this study are available in Zenodo at https://doi.org/10.5281/zenodo.16142636.
