# Supplementary material for: Invasion Potential of Calotropis procera (Aiton) W.T. Aiton and Xanthium strumarium L. in the Anthropocene of Ethiopia: Implications for Management
Source: Ecol Evol. 2025 Dec 9;15(12):e72577. doi: 10.1002/ece3.72577 (PMC12686969; doi:10.1002/ece3.72577)
Supplement: Supplementary file 1 — Appendix S1: ece372577‐sup‐0001‐AppendixS1.docx. [file ECE3-15-e72577-s001.docx]

**Invasion potential of *Calotropis procera* (Aiton) W.T Aiton and*Xanthium strumarium* L. in the Anthropocene of Ethiopia: Implication for management**

**Table S1** Occurrence locations of *Calotropis procera (Aiton) W.T Aiton*

| # | Species | Longitude | Latitude |
| --- | --- | --- | --- |
| 1 | *Calotropis procera* (Aiton) W.T Aiton | 38.353122 | 10.140737 |
| 2 | *Calotropis procera* (Aiton) W.T Aiton | 40.166667 | 9 |
| 3 | *Calotropis procera* (Aiton) W.T Aiton | 38.266667 | 10.083333 |
| 4 | *Calotropis procera* (Aiton) W.T Aiton | 39.083333 | 7.916667 |
| 5 | *Calotropis procera* (Aiton) W.T Aiton | 40.1 | 8.966667 |
| 6 | *Calotropis procera* (Aiton) W.T Aiton | 38.256703 | 14.084144 |
| 7 | *Calotropis procera* (Aiton) W.T Aiton | 38.316597 | 14.118279 |
| 8 | *Calotropis procera* (Aiton) W.T Aiton | 37.554945 | 5.229903 |
| 9 | *Calotropis procera* (Aiton) W.T Aiton | 40.271019 | 9.326993 |
| 10 | *Calotropis procera* (Aiton) W.T Aiton | 39.689429 | 13.374401 |
| 11 | *Calotropis procera* (Aiton) W.T Aiton | 39.722558 | 13.358682 |
| 12 | *Calotropis procera* (Aiton) W.T Aiton | 40.192345 | 9.07567 |
| 13 | *Calotropis procera* (Aiton) W.T Aiton | 39.101308 | 8.556221 |
| 14 | *Calotropis procera* (Aiton) W.T Aiton | 37.39906 | 5.610353 |
| 15 | *Calotropis procera* (Aiton) W.T Aiton | 37.414025 | 5.713968 |
| 16 | *Calotropis procera* (Aiton) W.T Aiton | 37.384197 | 7.379625 |
| 17 | *Calotropis procera* (Aiton) W.T Aiton | 38.924634 | 9.911977 |
| 18 | *Calotropis procera* (Aiton) W.T Aiton | 43.550793 | 5.93025 |
| 19 | *Calotropis procera* (Aiton) W.T Aiton | 40.489673 | 9.08406 |
| 20 | *Calotropis procera* (Aiton) W.T Aiton | 40.489673 | 9.145 |
| 21 | *Calotropis procera* (Aiton) W.T Aiton | 36.996567 | 5.21855 |
| 22 | *Calotropis procera* (Aiton) W.T Aiton | 44.24722 | 6.722836 |
| 23 | *Calotropis procera* (Aiton) W.T Aiton | 41.866667 | 9.616667 |
| 24 | *Calotropis procera* (Aiton) W.T Aiton | 41.6 | 9.6 |
| 25 | *Calotropis procera* (Aiton) W.T Aiton | 41.85 | 9.683333 |
| 26 | *Calotropis procera* (Aiton) W.T Aiton | 36.723642 | 4.828862 |
| 27 | *Calotropis procera* (Aiton) W.T Aiton | 38.837371 | 8.165204 |
| 28 | *Calotropis procera* (Aiton) W.T Aiton | 39.145708 | 8.593383 |
| 29 | *Calotropis procera* (Aiton) W.T Aiton | 37.754822 | 9.622625 |
| 30 | *Calotropis procera* (Aiton) W.T Aiton | 36.44173 | 5.772662 |
| 31 | *Calotropis procera* (Aiton) W.T Aiton | 38.728347 | 14.124528 |
| 32 | *Calotropis procera* (Aiton) W.T Aiton | 42.399914 | 9.028496 |
| 33 | *Calotropis procera* (Aiton) W.T Aiton | 40.62503 | 7.090961 |
| 34 | *Calotropis procera* (Aiton) W.T Aiton | 40.91188 | 13.320488 |
| 35 | *Calotropis procera* (Aiton) W.T Aiton | 38.965868 | 13.652173 |
| 36 | *Calotropis procera* (Aiton) W.T Aiton | 40.185654 | 9.032683 |
| 37 | *Calotropis procera* (Aiton) W.T Aiton | 38.191671 | 10.074246 |
| 38 | *Calotropis procera* (Aiton) W.T Aiton | 40.037439 | 13.874171 |
| 39 | *Calotropis procera* (Aiton) W.T Aiton | 35.075058 | 11.224505 |
| 40 | *Calotropis procera* (Aiton) W.T Aiton | 39.740759 | 13.348629 |
| 41 | *Calotropis procera* (Aiton) W.T Aiton | 38.705967 | 14.126389 |
| 42 | *Calotropis procera* (Aiton) W.T Aiton | 36.382378 | 4.758722 |
| 43 | *Calotropis procera* (Aiton) W.T Aiton | 37.629019 | 6.122275 |
| 44 | *Calotropis procera* (Aiton) W.T Aiton | 42.73333 | 9.61667 |
| 45 | *Calotropis procera* (Aiton) W.T Aiton | 41.63333 | 9.6 |
| 46 | *Calotropis procera* (Aiton) W.T Aiton | 42.3 | 9.21667 |
| 47 | *Calotropis procera* (Aiton) W.T Aiton | 39.754719 | 13.355508 |
| 48 | *Calotropis procera* (Aiton) W.T Aiton | 39.856158 | 13.434674 |
| 49 | *Calotropis procera* (Aiton) W.T Aiton | 39.025853 | 8.422863 |
| 50 | *Calotropis procera* (Aiton) W.T Aiton | 39.076039 | 8.510196 |
| 51 | *Calotropis procera* (Aiton) W.T Aiton | 39.143365 | 7.942505 |
| 52 | *Calotropis procera* (Aiton) W.T Aiton | 39.133065 | 7.957063 |
| 53 | *Calotropis procera* (Aiton) W.T Aiton | 41.883333 | 9.583333 |
| 54 | *Calotropis procera* (Aiton) W.T Aiton | 41.879117 | 9.532528 |
| 55 | *Calotropis procera* (Aiton) W.T Aiton | 41.858975 | 9.584964 |
| 56 | *Calotropis procera* (Aiton) W.T Aiton | 39.384367 | 8.618 |
| 57 | *Calotropis procera* (Aiton) W.T Aiton | 39.473467 | 8.675517 |
| 58 | *Calotropis procera* (Aiton) W.T Aiton | 39.573533 | 8.7363 |
| 59 | *Calotropis procera* (Aiton) W.T Aiton | 39.7813 | 8.8831 |
| 60 | *Calotropis procera* (Aiton) W.T Aiton | 39.318067 | 8.29685 |
| 61 | *Calotropis procera* (Aiton) W.T Aiton | 39.7263 | 8.84525 |
| 62 | *Calotropis procera* (Aiton) W.T Aiton | 39.745033 | 8.856617 |
| 63 | *Calotropis procera* (Aiton) W.T Aiton | 39.71055 | 8.82915 |
| 64 | *Calotropis procera* (Aiton) W.T Aiton | 39.54335 | 8.705433 |
| 65 | *Calotropis procera* (Aiton) W.T Aiton | 38.92665 | 8.277917 |
| 66 | *Calotropis procera* (Aiton) W.T Aiton | 39.29625 | 8.27135 |
| 67 | *Calotropis procera* (Aiton) W.T Aiton | 39.666317 | 8.792717 |
| 68 | *Calotropis procera* (Aiton) W.T Aiton | 39.76255 | 8.869367 |
| 69 | *Calotropis procera* (Aiton) W.T Aiton | 39.261967 | 8.223783 |
| 70 | *Calotropis procera* (Aiton) W.T Aiton | 39.690183 | 8.8105 |
| 71 | *Calotropis procera* (Aiton) W.T Aiton | 39.08455 | 4.611883 |
| 72 | *Calotropis procera* (Aiton) W.T Aiton | 39.157917 | 4.698467 |
| 73 | *Calotropis procera* (Aiton) W.T Aiton | 39.471233 | 5.035983 |
| 74 | *Calotropis procera* (Aiton) W.T Aiton | 39.675867 | 8.803333 |
| 75 | *Calotropis procera* (Aiton) W.T Aiton | 39.628617 | 8.775733 |
| 76 | *Calotropis procera* (Aiton) W.T Aiton | 40.656933 | 5.114433 |
| 77 | *Calotropis procera* (Aiton) W.T Aiton | 39.265617 | 4.792883 |
| 78 | *Calotropis procera* (Aiton) W.T Aiton | 39.307117 | 8.464633 |
| 79 | *Calotropis procera* (Aiton) W.T Aiton | 39.59195 | 8.751417 |
| 80 | *Calotropis procera* (Aiton) W.T Aiton | 39.911583 | 8.9065 |
| 81 | *Calotropis procera* (Aiton) W.T Aiton | 39.118367 | 8.591483 |
| 82 | *Calotropis procera* (Aiton) W.T Aiton | 40.2879 | 5.190783 |
| 83 | *Calotropis procera* (Aiton) W.T Aiton | 39.240017 | 4.762967 |
| 84 | *Calotropis procera* (Aiton) W.T Aiton | 39.646283 | 8.781283 |
| 85 | *Calotropis procera* (Aiton) W.T Aiton | 39.56225 | 8.725217 |
| 86 | *Calotropis procera* (Aiton) W.T Aiton | 39.244017 | 4.773733 |
| 87 | *Calotropis procera* (Aiton) W.T Aiton | 39.796017 | 8.891983 |
| 88 | *Calotropis procera* (Aiton) W.T Aiton | 39.328783 | 8.366883 |
| 89 | *Calotropis procera* (Aiton) W.T Aiton | 40.02495 | 8.911483 |
| 90 | *Calotropis procera* (Aiton) W.T Aiton | 39.460267 | 8.6765 |
| 91 | *Calotropis procera* (Aiton) W.T Aiton | 39.32595 | 8.345417 |
| 92 | *Calotropis procera* (Aiton) W.T Aiton | 40.5537 | 5.129833 |
| 93 | *Calotropis procera* (Aiton) W.T Aiton | 39.405983 | 4.886967 |
| 94 | *Calotropis procera* (Aiton) W.T Aiton | 39.058433 | 8.47305 |
| 95 | *Calotropis procera* (Aiton) W.T Aiton | 39.709183 | 8.817117 |
| 96 | *Calotropis procera* (Aiton) W.T Aiton | 39.527417 | 8.697833 |
| 97 | *Calotropis procera* (Aiton) W.T Aiton | 39.3744 | 8.609367 |
| 98 | *Calotropis procera* (Aiton) W.T Aiton | 39.012217 | 8.388783 |
| 99 | *Calotropis procera* (Aiton) W.T Aiton | 39.281183 | 8.253883 |
| 100 | *Calotropis procera* (Aiton) W.T Aiton | 39.445967 | 8.669917 |
| 101 | *Calotropis procera* (Aiton) W.T Aiton | 38.808783 | 8.137233 |
| 102 | *Calotropis procera* (Aiton) W.T Aiton | 39.119983 | 8.58105 |
| 103 | *Calotropis procera* (Aiton) W.T Aiton | 39.342733 | 8.5815 |
| 104 | *Calotropis procera* (Aiton) W.T Aiton | 39.209167 | 8.547933 |
| 105 | *Calotropis procera* (Aiton) W.T Aiton | 40.1617 | 8.985 |
| 106 | *Calotropis procera* (Aiton) W.T Aiton | 40.001883 | 8.9079 |
| 107 | *Calotropis procera* (Aiton) W.T Aiton | 39.311733 | 8.55515 |
| 108 | *Calotropis procera* (Aiton) W.T Aiton | 39.3186 | 8.4425 |
| 109 | *Calotropis procera* (Aiton) W.T Aiton | 39.4216 | 8.652467 |
| 110 | *Calotropis procera* (Aiton) W.T Aiton | 39.180817 | 8.555183 |
| 111 | *Calotropis procera* (Aiton) W.T Aiton | 39.331833 | 8.572567 |
| 112 | *Calotropis procera* (Aiton) W.T Aiton | 39.353517 | 8.5911 |
| 113 | *Calotropis procera* (Aiton) W.T Aiton | 39.301133 | 8.539017 |
| 114 | *Calotropis procera* (Aiton) W.T Aiton | 39.131183 | 8.581983 |
| 115 | *Calotropis procera* (Aiton) W.T Aiton | 39.237667 | 4.748883 |
| 116 | *Calotropis procera* (Aiton) W.T Aiton | 38.8573 | 8.828633 |
| 117 | *Calotropis procera* (Aiton) W.T Aiton | 39.638768 | 11.740374 |
| 118 | *Calotropis procera* (Aiton) W.T Aiton | 39.646725 | 11.555381 |
| 119 | *Calotropis procera* (Aiton) W.T Aiton | 39.171746 | 14.302822 |
| 120 | *Calotropis procera* (Aiton) W.T Aiton | 39.675782 | 11.648155 |
| 121 | *Calotropis procera* (Aiton) W.T Aiton | 39.8985 | 10.000867 |
| 122 | *Calotropis procera* (Aiton) W.T Aiton | 39.505936 | 13.15772 |
| 123 | *Calotropis procera* (Aiton) W.T Aiton | 39.526332 | 11.881107 |
| 124 | *Calotropis procera* (Aiton) W.T Aiton | 39.812983 | 10.873202 |
| 125 | *Calotropis procera* (Aiton) W.T Aiton | 39.620903 | 11.477215 |
| 126 | *Calotropis procera* (Aiton) W.T Aiton | 39.658008 | 11.594604 |
| 127 | *Calotropis procera* (Aiton) W.T Aiton | 38.630257 | 14.117004 |
| 128 | *Calotropis procera* (Aiton) W.T Aiton | 39.552858 | 13.449833 |
| 129 | *Calotropis procera* (Aiton) W.T Aiton | 39.94795 | 10.523083 |
| 130 | *Calotropis procera* (Aiton) W.T Aiton | 37.871126 | 13.334854 |
| 131 | *Calotropis procera* (Aiton) W.T Aiton | 37.855489 | 13.301085 |
| 132 | *Calotropis procera* (Aiton) W.T Aiton | 39.012939 | 14.231793 |
| 133 | *Calotropis procera* (Aiton) W.T Aiton | 37.628334 | 12.243037 |
| 134 | *Calotropis procera* (Aiton) W.T Aiton | 39.116875 | 11.947034 |
| 135 | *Calotropis procera* (Aiton) W.T Aiton | 39.769949 | 10.973395 |
| 136 | *Calotropis procera* (Aiton) W.T Aiton | 39.643515 | 11.850236 |
| 137 | *Calotropis procera* (Aiton) W.T Aiton | 39.683418 | 11.895057 |
| 138 | *Calotropis procera* (Aiton) W.T Aiton | 37.741129 | 12.068377 |
| 139 | *Calotropis procera* (Aiton) W.T Aiton | 38.536855 | 14.122422 |
| 140 | *Calotropis procera* (Aiton) W.T Aiton | 39.501704 | 13.525855 |
| 141 | *Calotropis procera* (Aiton) W.T Aiton | 37.291053 | 12.530462 |
| 142 | *Calotropis procera* (Aiton) W.T Aiton | 39.618019 | 11.451684 |
| 143 | *Calotropis procera* (Aiton) W.T Aiton | 37.853657 | 13.320522 |
| 144 | *Calotropis procera* (Aiton) W.T Aiton | 37.628772 | 12.230585 |
| 145 | *Calotropis procera* (Aiton) W.T Aiton | 39.598623 | 12.294925 |
| 146 | *Calotropis procera* (Aiton) W.T Aiton | 39.597057 | 12.229977 |
| 147 | *Calotropis procera* (Aiton) W.T Aiton | 39.607235 | 12.263427 |
| 148 | *Calotropis procera* (Aiton) W.T Aiton | 39.83605 | 9.911467 |
| 149 | *Calotropis procera* (Aiton) W.T Aiton | 39.585429 | 11.783003 |
| 150 | *Calotropis procera* (Aiton) W.T Aiton | 38.2293 | 14.068824 |
| 151 | *Calotropis procera* (Aiton) W.T Aiton | 38.86829 | 14.159868 |
| 152 | *Calotropis procera* (Aiton) W.T Aiton | 39.620345 | 11.835822 |
| 153 | *Calotropis procera* (Aiton) W.T Aiton | 37.10079 | 12.562573 |
| 154 | *Calotropis procera* (Aiton) W.T Aiton | 39.274192 | 14.311252 |
| 155 | *Calotropis procera* (Aiton) W.T Aiton | 36.984025 | 12.513351 |
| 156 | *Calotropis procera* (Aiton) W.T Aiton | 39.608719 | 12.243863 |
| 157 | *Calotropis procera* (Aiton) W.T Aiton | 39.668256 | 11.858868 |
| 158 | *Calotropis procera* (Aiton) W.T Aiton | 37.194383 | 12.528975 |
| 159 | *Calotropis procera* (Aiton) W.T Aiton | 39.576417 | 13.645504 |
| 160 | *Calotropis procera* (Aiton) W.T Aiton | 39.597954 | 12.318775 |
| 161 | *Calotropis procera* (Aiton) W.T Aiton | 39.074754 | 14.273545 |
| 162 | *Calotropis procera* (Aiton) W.T Aiton | 39.203646 | 14.320676 |
| 163 | *Calotropis procera* (Aiton) W.T Aiton | 37.31204 | 12.529908 |
| 164 | *Calotropis procera* (Aiton) W.T Aiton | 38.199261 | 13.992746 |
| 165 | *Calotropis procera* (Aiton) W.T Aiton | 39.222911 | 14.317002 |
| 166 | *Calotropis procera* (Aiton) W.T Aiton | 37.162186 | 12.52941 |
| 167 | *Calotropis procera* (Aiton) W.T Aiton | 39.13569 | 14.275819 |
| 168 | *Calotropis procera* (Aiton) W.T Aiton | 37.517204 | 12.423767 |
| 169 | *Calotropis procera* (Aiton) W.T Aiton | 38.440635 | 14.098507 |
| 170 | *Calotropis procera* (Aiton) W.T Aiton | 37.728565 | 12.038829 |
| 171 | *Calotropis procera* (Aiton) W.T Aiton | 37.687912 | 11.906105 |
| 172 | *Calotropis procera* (Aiton) W.T Aiton | 39.561106 | 12.407173 |
| 173 | *Calotropis procera* (Aiton) W.T Aiton | 39.034309 | 14.257955 |
| 174 | *Calotropis procera* (Aiton) W.T Aiton | 39.683921 | 11.876312 |
| 175 | *Calotropis procera* (Aiton) W.T Aiton | 37.709352 | 11.975395 |
| 176 | *Calotropis procera* (Aiton) W.T Aiton | 38.792088 | 14.399639 |
| 177 | *Calotropis procera* (Aiton) W.T Aiton | 37.441205 | 12.501058 |
| 178 | *Calotropis procera* (Aiton) W.T Aiton | 37.437173 | 12.535998 |
| 179 | *Calotropis procera* (Aiton) W.T Aiton | 38.810718 | 14.344462 |
| 180 | *Calotropis procera* (Aiton) W.T Aiton | 38.392198 | 14.072837 |
| 181 | *Calotropis procera* (Aiton) W.T Aiton | 38.819607 | 14.301612 |
| 182 | *Calotropis procera* (Aiton) W.T Aiton | 38.802626 | 14.36572 |
| 183 | *Calotropis procera* (Aiton) W.T Aiton | 37.629898 | 12.254394 |
| 184 | *Calotropis procera* (Aiton) W.T Aiton | 38.849155 | 14.152274 |
| 185 | *Calotropis procera* (Aiton) W.T Aiton | 38.343712 | 14.076347 |
| 186 | *Calotropis procera* (Aiton) W.T Aiton | 37.59553 | 5.1684 |
| 187 | *Calotropis procera* (Aiton) W.T Aiton | 36.96873 | 5.40007 |
| 188 | *Calotropis procera* (Aiton) W.T Aiton | 37.07128 | 5.33812 |
| 189 | *Calotropis procera* (Aiton) W.T Aiton | 37.4808 | 5.25704 |
| 190 | *Calotropis procera* (Aiton) W.T Aiton | 36.75212 | 5.47576 |
| 191 | *Calotropis procera* (Aiton) W.T Aiton | 37.14911 | 5.36215 |
| 192 | *Calotropis procera* (Aiton) W.T Aiton | 37.50333 | 5.23841 |
| 193 | *Calotropis procera* (Aiton) W.T Aiton | 37.1463 | 5.37338 |
| 194 | *Calotropis procera* (Aiton) W.T Aiton | 39.30922 | 8.28653 |
| 195 | *Calotropis procera* (Aiton) W.T Aiton | 37.34995 | 5.39111 |
| 196 | *Calotropis procera* (Aiton) W.T Aiton | 37.0977 | 5.34202 |
| 197 | *Calotropis procera* (Aiton) W.T Aiton | 37.4422 | 5.43445 |
| 198 | *Calotropis procera* (Aiton) W.T Aiton | 37.43134 | 5.72655 |
| 199 | *Calotropis procera* (Aiton) W.T Aiton | 37.1408 | 5.34536 |
| 200 | *Calotropis procera* (Aiton) W.T Aiton | 37.43373 | 5.50109 |
| 201 | *Calotropis procera* (Aiton) W.T Aiton | 36.83545 | 5.45873 |
| 202 | *Calotropis procera* (Aiton) W.T Aiton | 37.00002 | 5.36953 |
| 203 | *Calotropis procera* (Aiton) W.T Aiton | 38.85727 | 8.19031 |
| 204 | *Calotropis procera* (Aiton) W.T Aiton | 37.61818 | 5.13239 |
| 205 | *Calotropis procera* (Aiton) W.T Aiton | 37.56659 | 6.02797 |
| 206 | *Calotropis procera* (Aiton) W.T Aiton | 38.94426 | 8.29353 |
| 207 | *Calotropis procera* (Aiton) W.T Aiton | 36.85349 | 5.46211 |
| 208 | *Calotropis procera* (Aiton) W.T Aiton | 39.06569 | 8.48363 |
| 209 | *Calotropis procera* (Aiton) W.T Aiton | 37.28546 | 5.39738 |
| 210 | *Calotropis procera* (Aiton) W.T Aiton | 39.553397 | 8.712593 |
| 211 | *Calotropis procera* (Aiton) W.T Aiton | 40.196067 | 9.036657 |
| 212 | *Calotropis procera* (Aiton) W.T Aiton | 40.110082 | 8.953542 |
| 213 | *Calotropis procera* (Aiton) W.T Aiton | 39.809451 | 8.8962 |
| 214 | *Calotropis procera* (Aiton) W.T Aiton | 40.065305 | 8.930941 |
| 215 | *Calotropis procera* (Aiton) W.T Aiton | 39.983678 | 8.906917 |
| 216 | *Calotropis procera* (Aiton) W.T Aiton | 39.246724 | 8.53644 |
| 217 | *Calotropis procera* (Aiton) W.T Aiton | 39.978968 | 10.17791 |
| 218 | *Calotropis procera* (Aiton) W.T Aiton | 39.980778 | 10.33203 |
| 219 | *Calotropis procera* (Aiton) W.T Aiton | 38.47381 | 9.0103 |
| 220 | *Calotropis procera* (Aiton) W.T Aiton | 39.961755 | 10.343501 |
| 221 | *Calotropis procera* (Aiton) W.T Aiton | 39.972504 | 10.161396 |
| 222 | *Calotropis procera* (Aiton) W.T Aiton | 40.256748 | 11.248383 |
| 223 | *Calotropis procera* (Aiton) W.T Aiton | 39.988043 | 10.297367 |
| 224 | *Calotropis procera* (Aiton) W.T Aiton | 39.975597 | 10.098767 |
| 225 | *Calotropis procera* (Aiton) W.T Aiton | 40.529262 | 11.303541 |
| 226 | *Calotropis procera* (Aiton) W.T Aiton | 39.967272 | 10.138182 |
| 227 | *Calotropis procera* (Aiton) W.T Aiton | 40.680526 | 10.870888 |
| 228 | *Calotropis procera* (Aiton) W.T Aiton | 39.992618 | 10.223 |
| 229 | *Calotropis procera* (Aiton) W.T Aiton | 40.464572 | 9.754225 |
| 230 | *Calotropis procera* (Aiton) W.T Aiton | 40.755655 | 11.112559 |
| 231 | *Calotropis procera* (Aiton) W.T Aiton | 40.479401 | 9.772814 |
| 232 | *Calotropis procera* (Aiton) W.T Aiton | 40.724522 | 10.268563 |
| 233 | *Calotropis procera* (Aiton) W.T Aiton | 40.377235 | 11.259019 |
| 234 | *Calotropis procera* (Aiton) W.T Aiton | 40.566653 | 11.315379 |
| 235 | *Calotropis procera* (Aiton) W.T Aiton | 40.713817 | 10.433587 |
| 236 | *Calotropis procera* (Aiton) W.T Aiton | 39.963435 | 10.06805 |
| 237 | *Calotropis procera* (Aiton) W.T Aiton | 40.727975 | 11.324135 |
| 238 | *Calotropis procera* (Aiton) W.T Aiton | 40.214429 | 11.251751 |
| 239 | *Calotropis procera* (Aiton) W.T Aiton | 39.778566 | 10.95483 |
| 240 | *Calotropis procera* (Aiton) W.T Aiton | 40.309632 | 9.373596 |
| 241 | *Calotropis procera* (Aiton) W.T Aiton | 40.109817 | 11.220544 |
| 242 | *Calotropis procera* (Aiton) W.T Aiton | 42.617861 | 9.252999 |
| 243 | *Calotropis procera* (Aiton) W.T Aiton | 39.984984 | 10.207839 |
| 244 | *Calotropis procera* (Aiton) W.T Aiton | 40.747873 | 11.202129 |
| 245 | *Calotropis procera* (Aiton) W.T Aiton | 40.66815 | 11.344014 |
| 246 | *Calotropis procera* (Aiton) W.T Aiton | 40.388418 | 9.030203 |
| 247 | *Calotropis procera* (Aiton) W.T Aiton | 39.998539 | 10.234815 |
| 248 | *Calotropis procera* (Aiton) W.T Aiton | 40.348672 | 9.413447 |
| 249 | *Calotropis procera* (Aiton) W.T Aiton | 42.524715 | 9.229974 |
| 250 | *Calotropis procera* (Aiton) W.T Aiton | 40.418397 | 9.065683 |
| 251 | *Calotropis procera* (Aiton) W.T Aiton | 40.205454 | 9.054248 |
| 252 | *Calotropis procera* (Aiton) W.T Aiton | 42.483809 | 9.225122 |
| 253 | *Calotropis procera* (Aiton) W.T Aiton | 40.399381 | 9.04973 |
| 254 | *Calotropis procera* (Aiton) W.T Aiton | 39.859534 | 8.918952 |
| 255 | *Calotropis procera* (Aiton) W.T Aiton | 40.126879 | 8.962873 |
| 256 | *Calotropis procera* (Aiton) W.T Aiton | 40.75973 | 11.412498 |
| 257 | *Calotropis procera* (Aiton) W.T Aiton | 40.718097 | 10.637416 |
| 258 | *Calotropis procera* (Aiton) W.T Aiton | 40.43415 | 9.097569 |
| 259 | *Calotropis procera* (Aiton) W.T Aiton | 40.430911 | 9.635104 |
| 260 | *Calotropis procera* (Aiton) W.T Aiton | 40.391063 | 9.53392 |
| 261 | *Calotropis procera* (Aiton) W.T Aiton | 40.753789 | 10.516505 |
| 262 | *Calotropis procera* (Aiton) W.T Aiton | 40.450557 | 9.724282 |
| 263 | *Calotropis procera* (Aiton) W.T Aiton | 40.359397 | 11.254669 |
| 264 | *Calotropis procera* (Aiton) W.T Aiton | 39.896896 | 11.146422 |
| 265 | *Calotropis procera* (Aiton) W.T Aiton | 41.916945 | 9.481891 |
| 266 | *Calotropis procera* (Aiton) W.T Aiton | 40.734124 | 10.463877 |
| 267 | *Calotropis procera* (Aiton) W.T Aiton | 40.328943 | 9.401749 |
| 268 | *Calotropis procera* (Aiton) W.T Aiton | 39.409053 | 8.640078 |
| 269 | *Calotropis procera* (Aiton) W.T Aiton | 40.256911 | 9.014664 |
| 270 | *Calotropis procera* (Aiton) W.T Aiton | 40.390481 | 11.266818 |
| 271 | *Calotropis procera* (Aiton) W.T Aiton | 40.294814 | 9.346902 |
| 272 | *Calotropis procera* (Aiton) W.T Aiton | 40.145884 | 8.973454 |
| 273 | *Calotropis procera* (Aiton) W.T Aiton | 40.447774 | 9.705373 |
| 274 | *Calotropis procera* (Aiton) W.T Aiton | 42.638547 | 9.28519 |
| 275 | *Calotropis procera* (Aiton) W.T Aiton | 40.3359 | 9.0151 |
| 276 | *Calotropis procera* (Aiton) W.T Aiton | 40.3559 | 9.0186 |
| 277 | *Calotropis procera* (Aiton) W.T Aiton | 40.4395 | 9.1119 |
| 278 | *Calotropis procera* (Aiton) W.T Aiton | 40.5235 | 9.147 |
| 279 | *Calotropis procera* (Aiton) W.T Aiton | 40.549 | 9.1567 |
| 280 | *Calotropis procera* (Aiton) W.T Aiton | 40.582 | 9.173 |
| 281 | *Calotropis procera* (Aiton) W.T Aiton | 41.8559 | 9.6268 |
| 282 | *Calotropis procera* (Aiton) W.T Aiton | 40.1343 | 8.9967 |
| 283 | *Calotropis procera* (Aiton) W.T Aiton | 39.8978 | 8.909 |
| 284 | *Calotropis procera* (Aiton) W.T Aiton | 39.8749 | 8.919 |
| 285 | *Calotropis procera* (Aiton) W.T Aiton | 39.5022 | 8.6849 |
| 286 | *Calotropis procera* (Aiton) W.T Aiton | 39.2527 | 8.5057 |
| 287 | *Calotropis procera* (Aiton) W.T Aiton | 39.2185 | 8.5196 |
| 288 | *Calotropis procera* (Aiton) W.T Aiton | 39.1583 | 8.5875 |
| 289 | *Calotropis procera* (Aiton) W.T Aiton | 39.1165 | 8.6309 |
| 290 | *Calotropis procera* (Aiton) W.T Aiton | 42.3512 | 9.2318 |
| 291 | *Calotropis procera* (Aiton) W.T Aiton | 42.3413 | 9.2194 |
| 292 | *Calotropis procera* (Aiton) W.T Aiton | 42.3738 | 9.2012 |
| 293 | *Calotropis procera* (Aiton) W.T Aiton | 42.3602 | 9.2139 |
| 294 | *Calotropis procera* (Aiton) W.T Aiton | 42.3944 | 9.1804 |
| 295 | *Calotropis procera* (Aiton) W.T Aiton | 42.424 | 9.19 |
| 296 | *Calotropis procera* (Aiton) W.T Aiton | 42.4634 | 9.215 |
| 297 | *Calotropis procera* (Aiton) W.T Aiton | 42.4587 | 9.227 |
| 298 | *Calotropis procera* (Aiton) W.T Aiton | 42.5627 | 9.2319 |
| 299 | *Calotropis procera* (Aiton) W.T Aiton | 42.644 | 9.2956 |
| 300 | *Calotropis procera* (Aiton) W.T Aiton | 42.6739 | 9.3722 |
| 301 | *Calotropis procera* (Aiton) W.T Aiton | 42.757 | 9.3538 |
| 302 | *Calotropis procera* (Aiton) W.T Aiton | 42.799 | 9.3517 |
| 303 | *Calotropis procera* (Aiton) W.T Aiton | 43.6064 | 7.9758 |
| 304 | *Calotropis procera* (Aiton) W.T Aiton | 44.1117 | 6.6791 |
| 305 | *Calotropis procera* (Aiton) W.T Aiton | 44.7489 | 6.3844 |
| 306 | *Calotropis procera* (Aiton) W.T Aiton | 45.9575 | 6.9518 |
| 307 | *Calotropis procera* (Aiton) W.T Aiton | 37.433 | 5.74 |
| 308 | *Calotropis procera* (Aiton) W.T Aiton | 37.4425 | 5.6 |
| 309 | *Calotropis procera* (Aiton) W.T Aiton | 37.2168 | 5.396 |
| 310 | *Calotropis procera* (Aiton) W.T Aiton | 37.05 | 5.341 |
| 311 | *Calotropis procera* (Aiton) W.T Aiton | 37.0388 | 5.3535 |
| 312 | *Calotropis procera* (Aiton) W.T Aiton | 36.986 | 5.382 |
| 313 | *Calotropis procera* (Aiton) W.T Aiton | 36.8226 | 5.4612 |
| 314 | *Calotropis procera* (Aiton) W.T Aiton | 36.7699 | 5.4683 |
| 315 | *Calotropis procera* (Aiton) W.T Aiton | 36.7471 | 5.488 |
| 316 | *Calotropis procera* (Aiton) W.T Aiton | 36.458 | 5.7866 |
| 317 | *Calotropis procera* (Aiton) W.T Aiton | 45.6022 | 5.6792 |
| 318 | *Calotropis procera* (Aiton) W.T Aiton | 45.5227 | 5.6799 |
| 319 | *Calotropis procera* (Aiton) W.T Aiton | 45.4483 | 5.7295 |
| 320 | *Calotropis procera* (Aiton) W.T Aiton | 45.4936 | 5.8082 |
| 321 | *Calotropis procera* (Aiton) W.T Aiton | 45.7164 | 5.899 |
| 322 | *Calotropis procera* (Aiton) W.T Aiton | 45.7681 | 5.8195 |
| 323 | *Calotropis procera* (Aiton) W.T Aiton | 45.7678 | 5.8324 |
| 324 | *Calotropis procera* (Aiton) W.T Aiton | 45.9344 | 6.1372 |
| 325 | *Calotropis procera* (Aiton) W.T Aiton | 43.6123 | 8.221 |
| 326 | *Calotropis procera* (Aiton) W.T Aiton | 43.6063 | 8.1807 |
| 327 | *Calotropis procera* (Aiton) W.T Aiton | 43.6167 | 8.1847 |
| 328 | *Calotropis procera* (Aiton) W.T Aiton | 43.6299 | 8.1992 |
| 329 | *Calotropis procera* (Aiton) W.T Aiton | 43.6299 | 8.2208 |
| 330 | *Calotropis procera* (Aiton) W.T Aiton | 33.0063 | 7.8564 |

**Table S2** Occurrence locations of *Xanthium strumarium* L.

| # | Species | Longitude | Latitude |
| --- | --- | --- | --- |
| 1 | *Xanthium strumarium* L. | 39.3 | 8.266667 |
| 2 | *Xanthium strumarium* L. | 41.366667 | 9.533333 |
| 3 | *Xanthium strumarium* L. | 37.203632 | 6.433173 |
| 4 | *Xanthium strumarium* L. | 37.168217 | 11.70674 |
| 5 | *Xanthium strumarium* L. | 42.31667 | 9.21667 |
| 6 | *Xanthium strumarium* L. | 38.35 | 8.8 |
| 7 | *Xanthium strumarium* L. | 39.73333 | 11.08333 |
| 8 | *Xanthium strumarium* L. | 42.03333 | 9.4 |
| 9 | *Xanthium strumarium* L. | 41.63333 | 9.6 |
| 10 | *Xanthium strumarium* L. | 42.4 | 9.2 |
| 11 | *Xanthium strumarium* L. | 41.85 | 9.68333 |
| 12 | *Xanthium strumarium* L. | 41.18333 | 12.17833 |
| 13 | *Xanthium strumarium* L. | 38.508867 | 7.138917 |
| 14 | *Xanthium strumarium* L. | 39.532783 | 5.11125 |
| 15 | *Xanthium strumarium* L. | 39.631067 | 5.21795 |
| 16 | *Xanthium strumarium* L. | 38.565083 | 3.893317 |
| 17 | *Xanthium strumarium* L. | 38.24005 | 4.607633 |
| 18 | *Xanthium strumarium* L. | 39.472383 | 5.491983 |
| 19 | *Xanthium strumarium* L. | 39.52615 | 5.391733 |
| 20 | *Xanthium strumarium* L. | 38.197467 | 4.777717 |
| 21 | *Xanthium strumarium* L. | 39.039083 | 3.580883 |
| 22 | *Xanthium strumarium* L. | 39.382867 | 8.616633 |
| 23 | *Xanthium strumarium* L. | 38.280483 | 5.389167 |
| 24 | *Xanthium strumarium* L. | 38.1916 | 4.951883 |
| 25 | *Xanthium strumarium* L. | 39.345317 | 5.676817 |
| 26 | *Xanthium strumarium* L. | 39.647533 | 5.231683 |
| 27 | *Xanthium strumarium* L. | 38.47215 | 3.907317 |
| 28 | *Xanthium strumarium* L. | 39.261 | 5.810483 |
| 29 | *Xanthium strumarium* L. | 40.4671 | 5.14675 |
| 30 | *Xanthium strumarium* L. | 39.323183 | 5.751633 |
| 31 | *Xanthium strumarium* L. | 39.5793 | 5.3335 |
| 32 | *Xanthium strumarium* L. | 38.933467 | 3.666883 |
| 33 | *Xanthium strumarium* L. | 39.538117 | 5.359883 |
| 34 | *Xanthium strumarium* L. | 39.597583 | 5.322067 |
| 35 | *Xanthium strumarium* L. | 39.311733 | 8.55515 |
| 36 | *Xanthium strumarium* L. | 39.451883 | 4.931317 |
| 37 | *Xanthium strumarium* L. | 38.98145 | 8.333933 |
| 38 | *Xanthium strumarium* L. | 39.612243 | 11.503958 |
| 39 | *Xanthium strumarium* L. | 39.812983 | 10.873202 |
| 40 | *Xanthium strumarium* L. | 39.658008 | 11.594604 |
| 41 | *Xanthium strumarium* L. | 39.618019 | 11.451684 |
| 42 | *Xanthium strumarium* L. | 39.601649 | 13.804937 |
| 43 | *Xanthium strumarium* L. | 39.219733 | 11.948621 |
| 44 | *Xanthium strumarium* L. | 39.675782 | 11.648155 |
| 45 | *Xanthium strumarium* L. | 39.513188 | 13.548941 |
| 46 | *Xanthium strumarium* L. | 39.831387 | 10.811361 |
| 47 | *Xanthium strumarium* L. | 39.700946 | 11.086869 |
| 48 | *Xanthium strumarium* L. | 39.576417 | 13.645504 |
| 49 | *Xanthium strumarium* L. | 38.108415 | 13.49096 |
| 50 | *Xanthium strumarium* L. | 38.690906 | 14.116714 |
| 51 | *Xanthium strumarium* L. | 39.590642 | 12.412217 |
| 52 | *Xanthium strumarium* L. | 38.810718 | 14.344462 |
| 53 | *Xanthium strumarium* L. | 38.187639 | 13.85512 |
| 54 | *Xanthium strumarium* L. | 39.780695 | 10.936114 |
| 55 | *Xanthium strumarium* L. | 39.683418 | 11.895057 |
| 56 | *Xanthium strumarium* L. | 39.501704 | 13.525855 |
| 57 | *Xanthium strumarium* L. | 38.13015 | 13.552931 |
| 58 | *Xanthium strumarium* L. | 37.194383 | 12.528975 |
| 59 | *Xanthium strumarium* L. | 39.252702 | 14.325467 |
| 60 | *Xanthium strumarium* L. | 39.596109 | 11.800177 |
| 61 | *Xanthium strumarium* L. | 36.977609 | 12.516004 |
| 62 | *Xanthium strumarium* L. | 39.165716 | 11.94059 |
| 63 | *Xanthium strumarium* L. | 39.828497 | 10.748548 |
| 64 | *Xanthium strumarium* L. | 38.86829 | 14.159868 |
| 65 | *Xanthium strumarium* L. | 37.871126 | 13.334854 |
| 66 | *Xanthium strumarium* L. | 38.152837 | 13.632147 |
| 67 | *Xanthium strumarium* L. | 38.343712 | 14.076347 |
| 68 | *Xanthium strumarium* L. | 39.641975 | 11.381763 |
| 69 | *Xanthium strumarium* L. | 39.778537 | 10.954809 |
| 70 | *Xanthium strumarium* L. | 39.573622 | 12.376123 |
| 71 | *Xanthium strumarium* L. | 39.285996 | 14.289603 |
| 72 | *Xanthium strumarium* L. | 39.607235 | 12.263427 |
| 73 | *Xanthium strumarium* L. | 39.622562 | 11.543368 |
| 74 | *Xanthium strumarium* L. | 39.698592 | 12.432723 |
| 75 | *Xanthium strumarium* L. | 39.683269 | 11.2463 |
| 76 | *Xanthium strumarium* L. | 39.598866 | 13.773765 |
| 77 | *Xanthium strumarium* L. | 37.873119 | 13.277951 |
| 78 | *Xanthium strumarium* L. | 38.121207 | 13.533226 |
| 79 | *Xanthium strumarium* L. | 37.925137 | 13.353018 |
| 80 | *Xanthium strumarium* L. | 37.860502 | 13.285085 |
| 81 | *Xanthium strumarium* L. | 39.827583 | 9.8856 |
| 82 | *Xanthium strumarium* L. | 38.160625 | 13.674983 |
| 83 | *Xanthium strumarium* L. | 38.206556 | 13.740285 |
| 84 | *Xanthium strumarium* L. | 39.612716 | 12.415001 |
| 85 | *Xanthium strumarium* L. | 39.620903 | 11.477215 |
| 86 | *Xanthium strumarium* L. | 38.199261 | 13.992746 |
| 87 | *Xanthium strumarium* L. | 39.527208 | 13.326694 |
| 88 | *Xanthium strumarium* L. | 39.499863 | 13.144406 |
| 89 | *Xanthium strumarium* L. | 38.209293 | 13.824745 |
| 90 | *Xanthium strumarium* L. | 37.853657 | 13.320522 |
| 91 | *Xanthium strumarium* L. | 37.602701 | 12.318391 |
| 92 | *Xanthium strumarium* L. | 37.882275 | 13.259202 |
| 93 | *Xanthium strumarium* L. | 39.683921 | 11.876312 |
| 94 | *Xanthium strumarium* L. | 38.80761 | 14.333375 |
| 95 | *Xanthium strumarium* L. | 38.083436 | 13.479836 |
| 96 | *Xanthium strumarium* L. | 39.611012 | 11.519822 |
| 97 | *Xanthium strumarium* L. | 39.274192 | 14.311252 |
| 98 | *Xanthium strumarium* L. | 39.608719 | 12.243863 |
| 99 | *Xanthium strumarium* L. | 38.660592 | 14.109828 |
| 100 | *Xanthium strumarium* L. | 37.102829 | 11.403293 |
| 101 | *Xanthium strumarium* L. | 39.515934 | 13.259485 |
| 102 | *Xanthium strumarium* L. | 39.797133 | 9.853567 |
| 103 | *Xanthium strumarium* L. | 37.978904 | 13.383112 |
| 104 | *Xanthium strumarium* L. | 39.655632 | 11.67504 |
| 105 | *Xanthium strumarium* L. | 38.87512 | 14.171797 |
| 106 | *Xanthium strumarium* L. | 38.954896 | 14.199714 |
| 107 | *Xanthium strumarium* L. | 38.183265 | 13.926694 |
| 108 | *Xanthium strumarium* L. | 39.52824 | 13.343819 |
| 109 | *Xanthium strumarium* L. | 37.18527 | 12.541475 |
| 110 | *Xanthium strumarium* L. | 39.646558 | 12.416938 |
| 111 | *Xanthium strumarium* L. | 37.766945 | 12.107965 |
| 112 | *Xanthium strumarium* L. | 38.035661 | 13.419322 |
| 113 | *Xanthium strumarium* L. | 39.673828 | 12.42455 |
| 114 | *Xanthium strumarium* L. | 39.011296 | 11.960038 |
| 115 | *Xanthium strumarium* L. | 38.880296 | 14.194339 |
| 116 | *Xanthium strumarium* L. | 37.212095 | 11.438435 |
| 117 | *Xanthium strumarium* L. | 38.2293 | 14.068824 |
| 118 | *Xanthium strumarium* L. | 39.568742 | 13.634438 |
| 119 | *Xanthium strumarium* L. | 37.030993 | 12.517967 |
| 120 | *Xanthium strumarium* L. | 38.314138 | 14.117802 |
| 121 | *Xanthium strumarium* L. | 38.466443 | 14.113015 |
| 122 | *Xanthium strumarium* L. | 37.673905 | 12.185338 |
| 123 | *Xanthium strumarium* L. | 39.272205 | 14.330132 |
| 124 | *Xanthium strumarium* L. | 37.899726 | 13.339769 |
| 125 | *Xanthium strumarium* L. | 37.855489 | 13.301085 |
| 126 | *Xanthium strumarium* L. | 38.196373 | 13.735632 |
| 127 | *Xanthium strumarium* L. | 37.965882 | 13.373467 |
| 128 | *Xanthium strumarium* L. | 37.329033 | 11.565594 |
| 129 | *Xanthium strumarium* L. | 37.995488 | 13.393608 |
| 130 | *Xanthium strumarium* L. | 37.282198 | 12.531771 |
| 131 | *Xanthium strumarium* L. | 37.628772 | 12.230585 |
| 132 | *Xanthium strumarium* L. | 37.722834 | 12.168707 |
| 133 | *Xanthium strumarium* L. | 37.86059 | 5.13255 |
| 134 | *Xanthium strumarium* L. | 37.58493 | 5.1986 |
| 135 | *Xanthium strumarium* L. | 39.28052 | 8.51036 |
| 136 | *Xanthium strumarium* L. | 37.44191 | 5.70058 |
| 137 | *Xanthium strumarium* L. | 36.631 | 5.69988 |
| 138 | *Xanthium strumarium* L. | 37.50333 | 5.23841 |
| 139 | *Xanthium strumarium* L. | 39.22383 | 7.62816 |
| 140 | *Xanthium strumarium* L. | 37.4422 | 5.43445 |
| 141 | *Xanthium strumarium* L. | 38.33718 | 4.80724 |
| 142 | *Xanthium strumarium* L. | 37.44365 | 5.58874 |
| 143 | *Xanthium strumarium* L. | 37.66478 | 6.15517 |
| 144 | *Xanthium strumarium* L. | 38.50041 | 7.02767 |
| 145 | *Xanthium strumarium* L. | 37.56659 | 6.02797 |
| 146 | *Xanthium strumarium* L. | 37.71922 | 5.03631 |
| 147 | *Xanthium strumarium* L. | 37.56418 | 5.2212 |
| 148 | *Xanthium strumarium* L. | 37.69028 | 6.17667 |
| 149 | *Xanthium strumarium* L. | 37.73669 | 4.92456 |
| 150 | *Xanthium strumarium* L. | 37.64934 | 5.1223 |
| 151 | *Xanthium strumarium* L. | 37.4941 | 5.88942 |
| 152 | *Xanthium strumarium* L. | 37.43373 | 5.50109 |
| 153 | *Xanthium strumarium* L. | 37.53881 | 5.9919 |
| 154 | *Xanthium strumarium* L. | 37.45194 | 5.66077 |
| 155 | *Xanthium strumarium* L. | 37.79422 | 6.53073 |
| 156 | *Xanthium strumarium* L. | 37.43872 | 5.34641 |
| 157 | *Xanthium strumarium* L. | 37.56479 | 6.10037 |
| 158 | *Xanthium strumarium* L. | 36.6148 | 5.7307 |
| 159 | *Xanthium strumarium* L. | 38.36158 | 4.80211 |
| 160 | *Xanthium strumarium* L. | 37.43134 | 5.72655 |
| 161 | *Xanthium strumarium* L. | 37.75465 | 6.25224 |
| 162 | *Xanthium strumarium* L. | 37.8103 | 6.54183 |
| 163 | *Xanthium strumarium* L. | 37.61818 | 5.13239 |
| 164 | *Xanthium strumarium* L. | 37.5682 | 6.07357 |
| 165 | *Xanthium strumarium* L. | 37.46917 | 5.8518 |
| 166 | *Xanthium strumarium* L. | 37.80976 | 6.65639 |
| 167 | *Xanthium strumarium* L. | 37.43286 | 5.57883 |
| 168 | *Xanthium strumarium* L. | 37.76655 | 6.49024 |
| 169 | *Xanthium strumarium* L. | 37.43712 | 5.75706 |
| 170 | *Xanthium strumarium* L. | 37.75606 | 6.41758 |
| 171 | *Xanthium strumarium* L. | 37.59553 | 5.1684 |
| 172 | *Xanthium strumarium* L. | 39.33044 | 8.38768 |
| 173 | *Xanthium strumarium* L. | 37.50743 | 5.91565 |
| 174 | *Xanthium strumarium* L. | 37.77743 | 6.27169 |
| 175 | *Xanthium strumarium* L. | 37.75696 | 6.47361 |
| 176 | *Xanthium strumarium* L. | 38.11521 | 4.8877 |
| 177 | *Xanthium strumarium* L. | 37.44238 | 5.62823 |
| 178 | *Xanthium strumarium* L. | 37.65248 | 6.13679 |
| 179 | *Xanthium strumarium* L. | 37.77819 | 6.51638 |
| 180 | *Xanthium strumarium* L. | 37.75053 | 6.4409 |
| 181 | *Xanthium strumarium* L. | 37.83783 | 6.59208 |
| 182 | *Xanthium strumarium* L. | 37.45847 | 5.80841 |
| 183 | *Xanthium strumarium* L. | 40.713817 | 10.433587 |
| 184 | *Xanthium strumarium* L. | 39.808183 | 10.830857 |
| 185 | *Xanthium strumarium* L. | 39.978968 | 10.17791 |
| 186 | *Xanthium strumarium* L. | 40.529262 | 11.303541 |
| 187 | *Xanthium strumarium* L. | 39.221533 | 8.535667 |
| 188 | *Xanthium strumarium* L. | 39.992618 | 10.223 |
| 189 | *Xanthium strumarium* L. | 39.961755 | 10.343501 |
| 190 | *Xanthium strumarium* L. | 39.27945 | 8.549167 |
| 191 | *Xanthium strumarium* L. | 42.585734 | 9.23365 |
| 192 | *Xanthium strumarium* L. | 39.909596 | 10.667648 |
| 193 | *Xanthium strumarium* L. | 42.195052 | 9.26091 |
| 194 | *Xanthium strumarium* L. | 42.41676 | 9.191655 |
| 195 | *Xanthium strumarium* L. | 42.232112 | 9.248428 |
| 196 | *Xanthium strumarium* L. | 39.881627 | 10.704073 |
| 197 | *Xanthium strumarium* L. | 37.4 | 11.6 |
| 198 | *Xanthium strumarium* L. | 42.4 | 9.18 |
| 199 | *Xanthium strumarium* L. | 39.3 | 8.266667 |
| 200 | *Xanthium strumarium* L. | 41.366667 | 9.533333 |
| 201 | *Xanthium strumarium* L. | 37.203632 | 6.433173 |
| 202 | *Xanthium strumarium* L. | 37.168217 | 11.70674 |


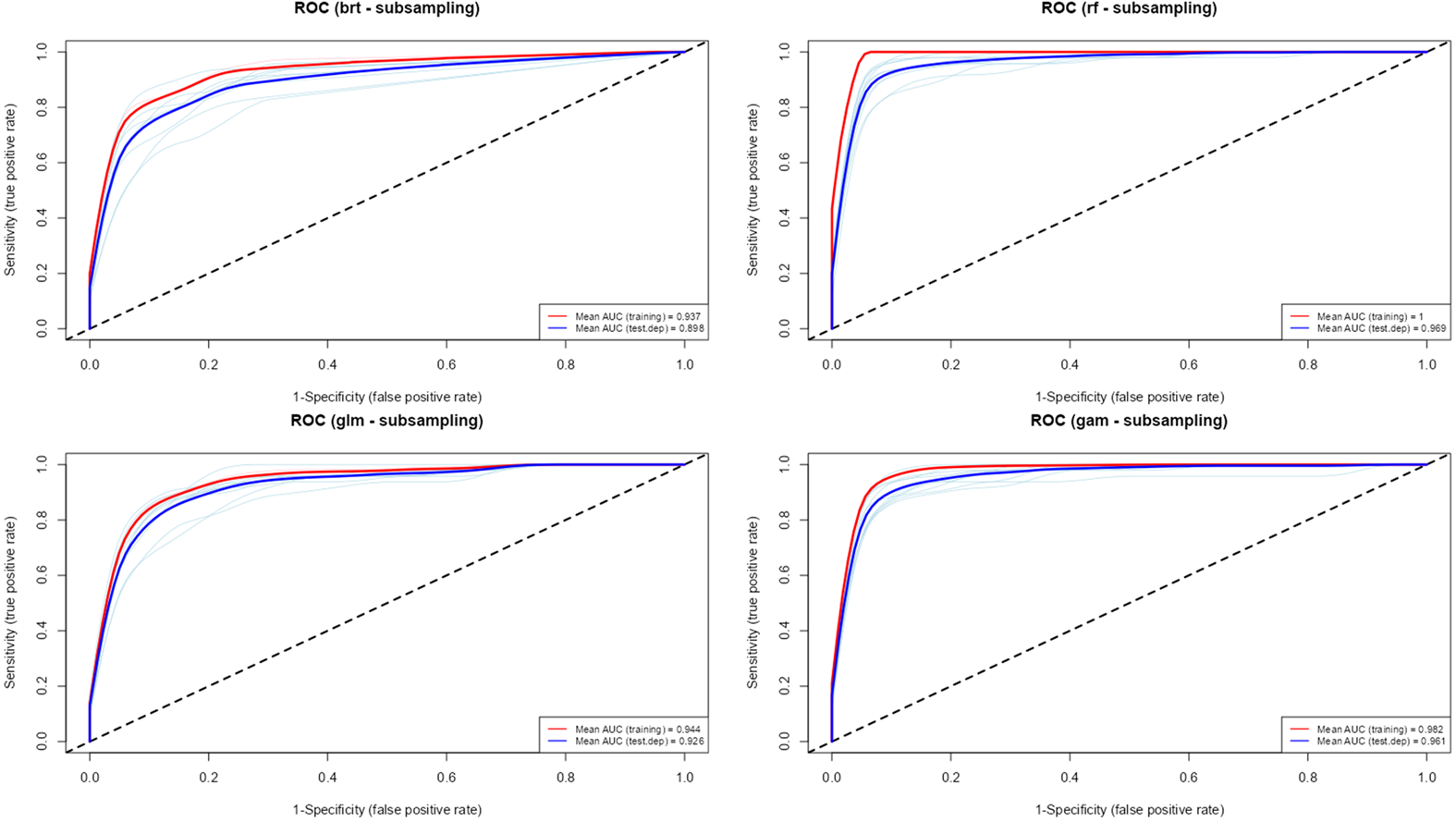


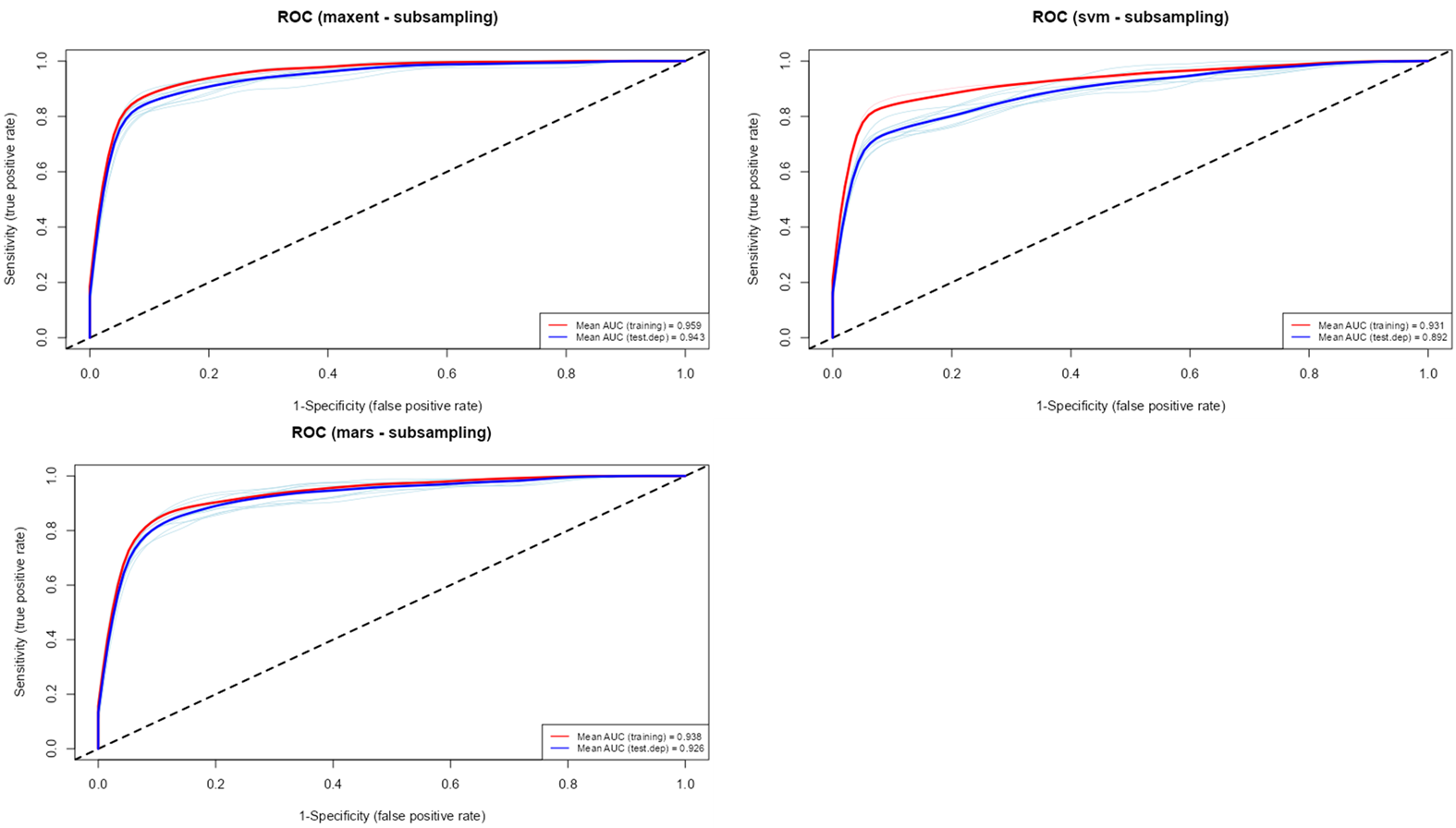


**figure S1** The Receiver Operating Characteristic (ROC) Curve of *Calotropis procera* of mean AUC for training and testing with 10 replications.


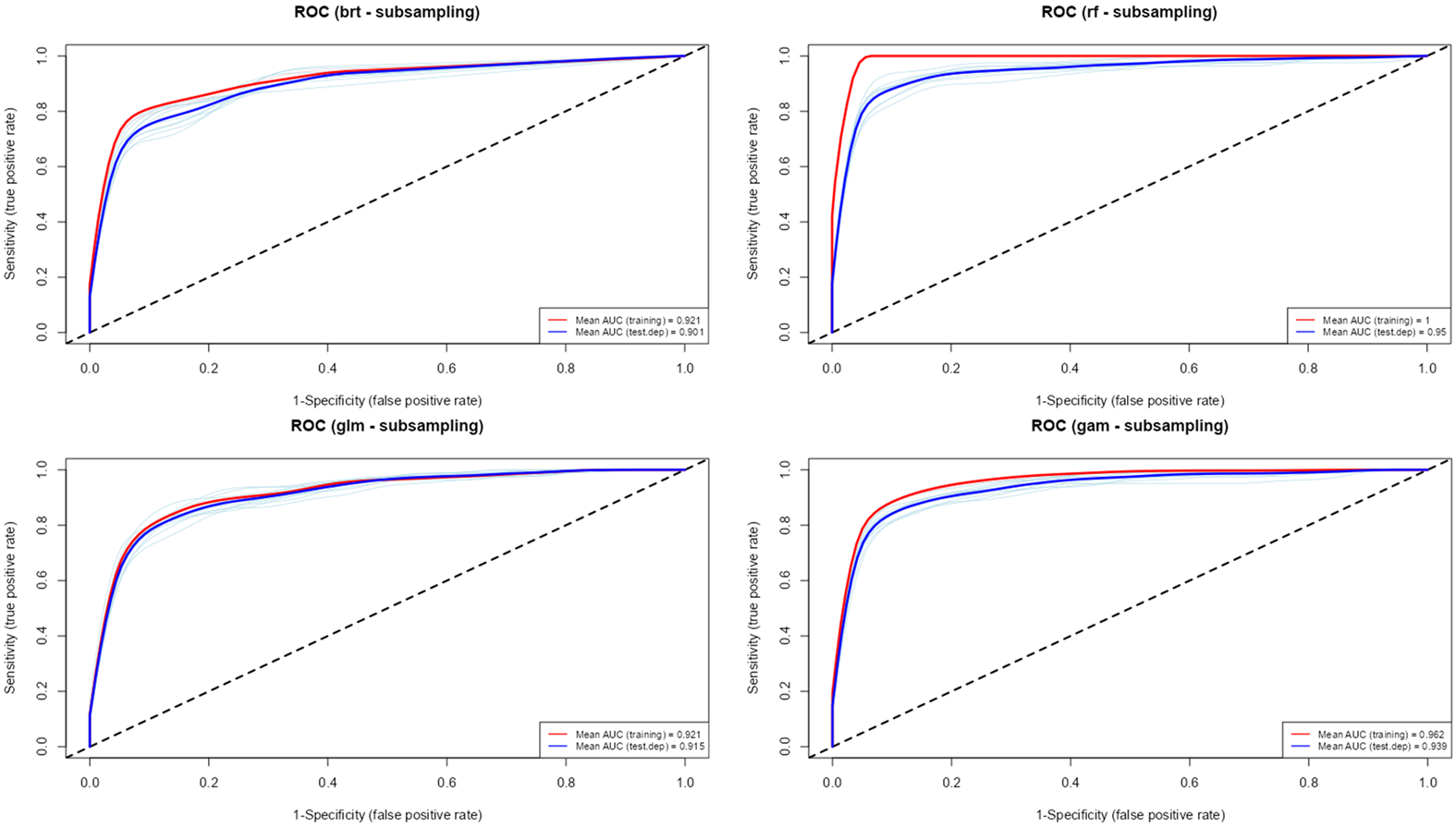


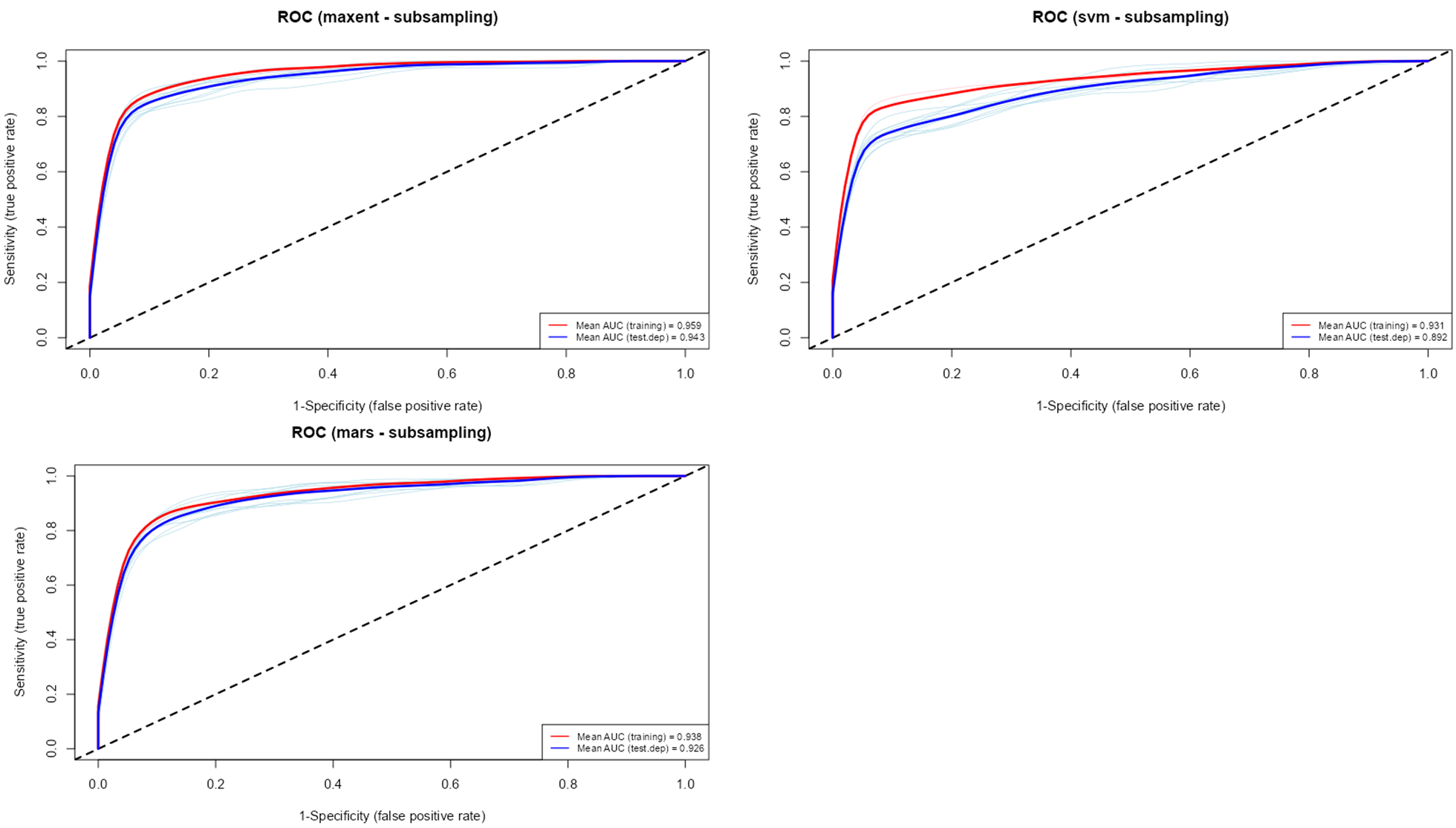


**figure S2** The Receiver Operating Characteristic (ROC) Curve of *Xanthium strumarium,* which indicates the mean AUC for training and testing with 10 replications.


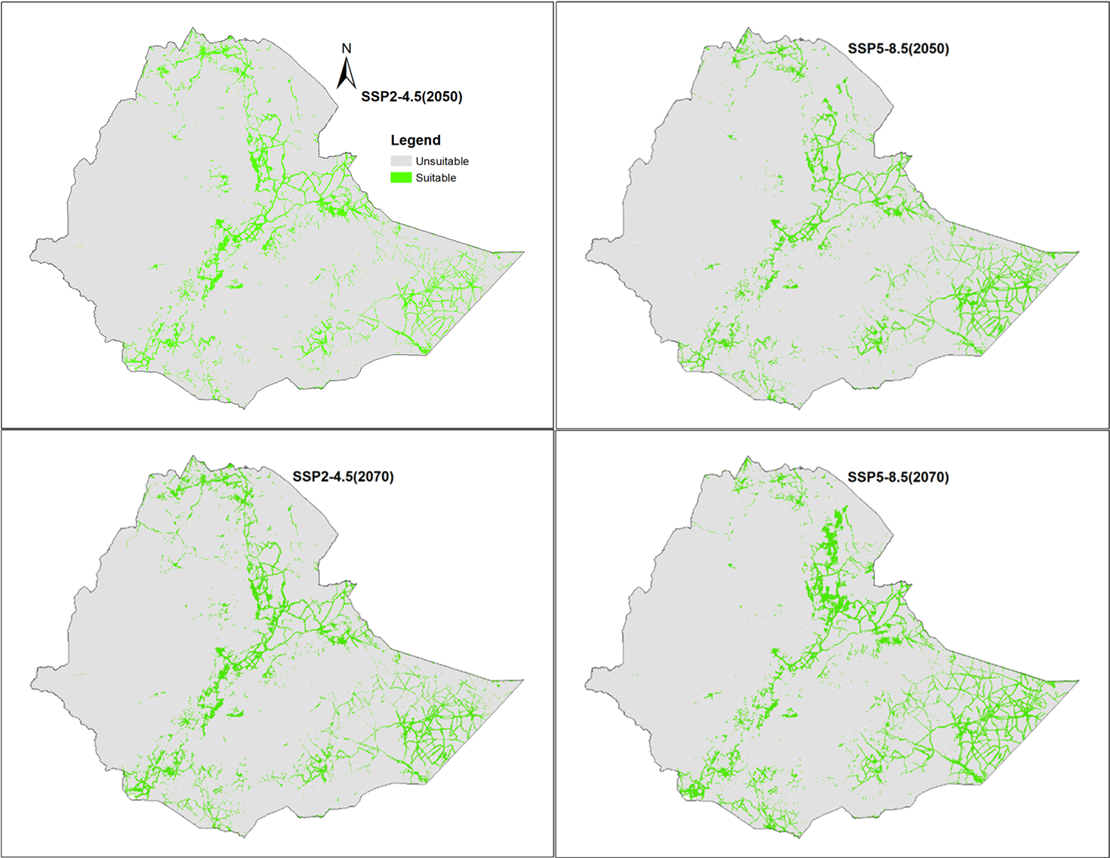


**figure S3** The distribution and extent of habitat suitability for *C. procera* were produced using seven different algorisms with ten replicated ensemble maps under future climate scenarios


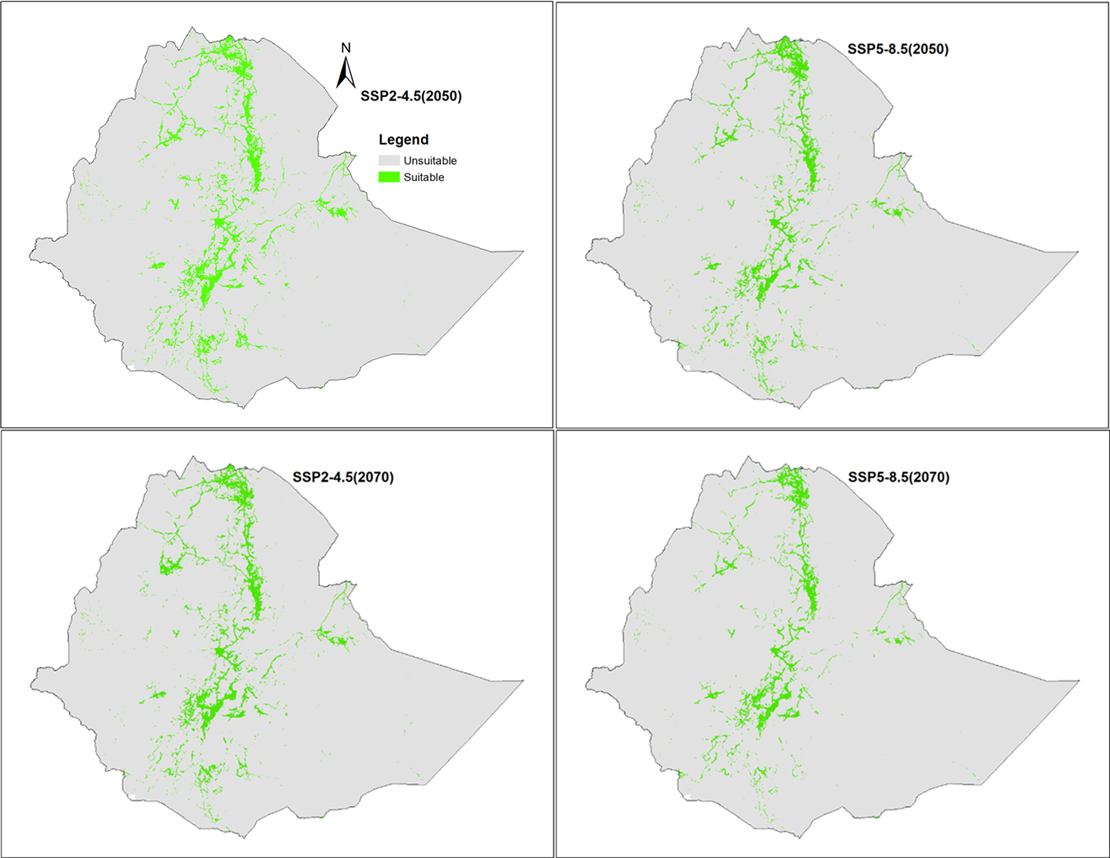


**figure S4** The distribution and extent of habitat suitability for *X. strumarium* were produced using seven different algorithms with ten replicated ensemble maps under future climate scenarios
